# Identification of immune-related biomarkers for intracerebral hemorrhage diagnosis based on RNA sequencing and machine learning

**DOI:** 10.3389/fimmu.2024.1421942

**Published:** 2024-08-30

**Authors:** Congxia Bai, Xinran Liu, Fengjuan Wang, Yingying Sun, Jing Wang, Jing Liu, Xiaoyan Hao, Lei Zhou, Yu Yuan, Jiayun Liu

**Affiliations:** ^1^ Department of Clinical Laboratory Medicine, Xijing Hospital, Fourth Military Medical University, Xi’an, China; ^2^ State Key Laboratory of Cardiovascular Disease, Fuwai Hospital, National Center for Cardiovascular Diseases, Chinese Academy of Medical Sciences and Peking Union Medical College, Beijing, China; ^3^ Neurosurgery, Affiliated Hospital of Hebei University, Baoding, Hebei, China

**Keywords:** intracerebral hemorrhage, RNA sequencing, immune cells, biomarkers, machine learning

## Abstract

**Background:**

Intracerebral hemorrhage (ICH) is a severe stroke subtype with high morbidity, disability, and mortality rates. Currently, no biomarkers for ICH are available for use in clinical practice. We aimed to explore the roles of RNAs in ICH pathogenesis and identify potential diagnostic biomarkers.

**Methods:**

We collected 233 individual blood samples from two independent cohorts, including 64 patients with ICH, 59 patients with ischemic stroke (IS), 60 patients with hypertension (HTN) and 50 healthy controls (CTRL) for RNA sequencing. Differentially expressed genes (DEGs) analysis, gene set enrichment analysis (GSEA), and weighted correlation network analysis (WGCNA) were performed to identify ICH-specific modules. The immune cell composition was evaluated with ImmuneCellAI. Multiple machine learning algorithms to select potential biomarkers for ICH diagnosis, and further validated by quantitative real-time polymerase chain reaction (RT−PCR). Receiver operating characteristic (ROC) curve analysis and decision curve analysis (DCA) were performed to evaluate the diagnostic value of the signature for ICH. Finally, we generated M1 and M2 macrophages to investigate the expression of candidate genes.

**Results:**

In both cohorts, 519 mRNAs and 131 lncRNAs were consistently significantly differentially expressed between ICH patients and HTN controls. Gene function analysis suggested that immune system processes may be involved in ICH pathology. ImmuneCellAI analysis revealed that the abundances of 11 immune cell types were altered after ICH in both cohorts. WGCNA and GSEA identified 18 immune-related DEGs. Multiple algorithms identified an RNA panel (CKAP4, BCL6, TLR8) with high diagnostic value for discriminating ICH patients from HTN controls, CTRLs and IS patients (AUCs: 0.93, 0.95 and 0.82; sensitivities: 81.3%, 84.4% and 75%; specificities: 100%, 96% and 79.7%, respectively). Additionally, CKAP4 and TLR8 mRNA and protein levels decreased in RAW264.7 M1 macrophages and increased in RAW264.7 M2 macrophages, while BCL6 expression increased in M1 macrophages but not in M2 macrophages, which may provide potential therapeutic targets for ICH.

**Conclusions:**

This study demonstrated that the expression levels of lncRNAs and mRNAs are associated with ICH, and an RNA panel (CKAP4, BCL6, TLR8) was developed as a potential diagnostic tool for distinguishing ICH from IS and controls, which could provide useful insight into ICH diagnosis and pathogenesis.

## Introduction

Stroke remains the second leading cause of death worldwide and has been regarded as a global health burden at both the individual and societal levels (1). The estimated number of incident strokes was 13.7 million in 2016, approximately 87% of which were ischemic stroke (IS) (2). In the same year, IS and intracerebral hemorrhage (ICH) accounted for 2.7 million and 2.8 million deaths, respectively (3). ICH accounts for approximately 23.8% of strokes in China, with a mortality rate of 67.9%, which is higher than that of IS (4). Hypertension (HTN) is the most common risk factor for stroke (5, 6), accounting for approximately 65% of all ICHs (7, 8). Although effective HTN management has reduced the incidence of ICH in some high-income countries, the incidence and prevalence of ICH have increased in China (5), and HTN remains the greatest risk factor for ICH. Currently, the diagnosis of stroke depends on neuroimaging techniques, and clinicians often face diagnostic challenges in distinguishing between ICH and acute IS because clinical findings can be vague and neuroimaging (especially magnetic resonance imaging) is difficult. Early identification of patients with acute IS is essential because reperfusion therapy can be administered soon after stroke onset, which is very important for achieving recovery and a good prognosis in IS patients (9). In recent years, the detection of biomarkers has become important for assisting in the early diagnosis of stroke. However, none of these methods has proven to be completely reliable or has become a clinical standard. The currently used stroke biomarkers are limited by insufficient specificity, difficulties related to detection and acquisition, a detection time beyond the thrombolysis window, and establishment on the basis of a small sample size (10, 11). Thus, identifying potential diagnostic biomarkers and understanding the pathophysiological mechanisms underlying the development of ICH are essential.

Blood samples are easily accessible and acceptable for patients, which makes them attractive for biomarker discovery and validation (12). Emerging evidence has revealed that peripheral blood cells play vital roles in the neurological injury caused by ICH and that global messenger RNA (mRNA) and noncoding RNA (ncRNA) expression profiles are altered rapidly in the blood after ICH (13–15). ncRNAs are involved in various biological processes associated with stroke (16, 17) and are potential biomarkers and therapeutic tools. ncRNAs include long noncoding RNAs (lncRNAs), circular RNAs (circRNAs) and microRNAs (miRNAs), which are involved in the regulation of transcription and translation (18). Our previous studies focused on identifying circRNAs as potential biomarkers for ICH diagnosis (19, 20). In this study, we aimed to investigate the expression profiles of lncRNAs and mRNAs by using RNA sequencing data from two independent cohorts and investigated the potential functions of the identified RNAs via gene ontology (GO) and pathway analyses. We aimed to explore RNA expression profiles and functions to identify specific mRNAs and lncRNAs as potential biomarkers for the diagnosis of ICH, which might provide useful insight into the pathogenesis of ICH and a more effective diagnostic tool.

## Materials and methods

### Study subjects

The participants enrolled in this study were described in our previous study (19, 20). The study protocol was reviewed and approved by the Human Ethics Committee, Fuwai Hospital (Approval No. 2016-732), and the study was conducted in accordance with the principles of Good Clinical Practice and the Declaration of Helsinki. Written informed consent was obtained from all study participants or their legal proxies.

In brief, a total of 273 individuals, including individuals with ICH (n=84), IS (n = 59), or HTN (n= 60) and age-matched healthy controls (CTRLs, n=70), were recruited from three individual cohorts between 2014 and 2024. In the first cohort, 160 individuals (44 ICH patients, 43 IS patients, 42 HTN controls and 31 CTRLs) from Cangzhou Central Hospital who were enrolled between 2014 and 2017 composed the discovery phase. In the second cohort, 20 consecutive ICH patients from Hebei Baoding Hospital, another 18 HTN controls and 16 IS patients from the General Hospital of Ningxia Medical University, and 19 CTRLs from Tsinghua University Hospital were enrolled between 2017 and 2019. Additionally, 20 ICH patients and 20 CTRLs from Xijing Hospital were enrolled as an independent validation cohort between 2023 and 2024. ICH and IS were diagnosed by professional neurologists on the basis of medical history and exams and confirmed by computed tomography (CT) or magnetic resonance imaging (MRI) (21). HTN controls with simple HTN but without a history of previous stroke or cardiovascular events were selected as the HTN control group, and the CTRLs group was matched for age, sex, and vascular risk factors, including diabetes mellitus, hyperlipidemia, and smoking and drinking status. Information on demographic and clinical characteristics was obtained through face−to-face surveys and by checking hospital records or medical examination records (**Tables 1**, **2**). The exclusion criteria included autoimmune diseases, cardiac disease, liver diseases, renal diseases, cancer, and a history of previous stroke or IS with hemorrhagic transformation.

**Table 1:** Demographics and characteristics of the discovery cohorts.

	CTRL (n=31)	HTN (n = 42)	ICH (n = 44)	IS (n = 43)	*P*-value
Age, y	58.9 ± 5.3	57.5 ± 6.2	55.9 ± 7.2	57.4 ± 5.5	0.264
Men, %	17 (54.8)	18 (42.8)	24 (54.5)	21 (48.8)	0.719
BMI, kg/m^2^	24.8 ± 2.9	24.5 ± 3.6	26.1 ± 6.6	27.6 ± 6.9	0.068
SBP, mmHg	125.7 ± 10.1	138.3 ± 12.7	137.4 ± 17.6	138.6 ± 13.6	0.995
DBP, mmHg	79.2 ± 4.3	91.7 ± 19.1	87.9 ± 10.7	91.8 ± 16.6	0.624
HDL-C, mmol/L	1.4 ± 0.3	1.4 ± 0.3	1.1 ± 0.3	1.1 ± 0.2	0.955
LDL-C, mmol/L	2.9 ± 0.7	2.9 ± 0.7	2.4 ± 0.8	2.3 ± 0.8	0.997
TC, mmol/L	5.5 ± 1.0	5.6 ± 1.0	4.5 ± 1.0	4.5 ± 1.0	0.934
TG, mmol/L	1.4 ± 0.8	1.7 ± 1.0	1.5 ± 0.9	1.6 ± 0.6	0.269
Glucose, mmol/L	6.0 ± 1.8	6.0 ± 1.8	6.3 ± 1.6	5.9 ± 1.3	0.317
Smoking, %					0.963
Never	19 (61.3)	25 (59.5)	28 (63.7)	26 (60.5)	
Former	4 (12.9)	5 (11.9)	5 (13.6)	8 (18.6)	
Current	8 (25.8)	12 (28.6)	11 (22.7)	9 (20.9)	
Drinking, %					0.517
Nondrinker	20 (64.5)	26 (61.9)	28 (63.6)	27 (62.8)	
Drinker	11 (35.5)	16 (38.1)	16 (36.4)	16 (37.2)	

Data is expressed as mean ± standard deviation or n (%). BMI, Body mass index; SBP, Systolic blood pressure; DBP, Diastolic blood pressure; TC, Total cholesterol; TG, Triacylglycerol; HDL-C, High-density lipoprotein cholesterol; LDL-C, Low-density lipoprotein cholesterol; GLU, Glucose; ICH, Intracerebral hemorrhage; IS, ischemic stroke; HTN, hypertension; CTRL, healthy control. Statistical comparisons for percentages were performed using chi-square test. Comparisons between means or medians were performed using a One-way ANOVA.

**Table 2:** Demographics and characteristics of the validation cohorts.

	CTRL (n = 19)	HTN (n=18)	ICH (n = 20)	IS (n = 16)	*P*-value
Age, y	57.2 ± 7.0	56.2 ± 7.2	56.7 ± 7.1	57.2 ± 7.7	0.952
Men, %	10 (52.6)	11 (60)	10 (50)	8 (50)	0.998
BMI, kg/m^2^	24.9 ± 2.4	24.9 ± 2.4	25.8 ± 6.8	25.0 ± 2.6	0.970
SBP, mmHg	120.3 ± 9.7	133.1 ± 18.9	171.2 ± 25.7	150.6 ± 19.4	0.999
DBP, mmHg	77.6 ± 9.1	91.7 ± 12.4	103.7 ± 13.3	89.3 ± 13.9	0.927
HDL-C, mmol/L	1.3 ± 0.3	1.2 ± 0.3	0.9 ± 0.5	1.0 ± 0.3	0.089
LDL-C, mmol/L	2.9 ± 0.9	2.9 ± 0.94	2.8 ± 0.8	2.7 ± 0.9	0.940
TC, mmol/L	4.5 ± 1.0	4.8 ± 2.0	4.3 ± 0.9	4.9 ± 1.3	0.269
TG, mmol/L	1.2 ± 0.5	1.9 ± 0.8	1.4 ± 0.6	2.3 ± 1.5	0.058
Glucose, mmol/L	5.3 ± 0.6	5.2 ± 0.5	5.5 ± 1.7	6.0 ± 1.1	0.109
Smoking, %					0.288
Never	13 (68.4)	12 (66.7)	14 (70)	11 (68.7)	
Former	3 (15.8)	2 (11.1)	2 (10)	2 (12.5)	
Current	3 (15.8)	4 (22.2)	4 (20)	3 (18.8)	
Drinking, %					0.090
Nondrinker	11 (57.9)	12 (66.7)	12 (60)	10 (62.5)	
Drinker	8 (42.1)	6 (33.3)	8 (40)	6 (37.5)	

Data is expressed as mean ± standard deviation or n (%). BMI, Body mass index; SBP, Systolic blood pressure; DBP, Diastolic blood pressure; TC, Total cholesterol; TG, Triacylglycerol; HDL-C, High-density lipoprotein cholesterol; LDL-C, Low-density lipoprotein cholesterol; GLU, Glucose; ICH, Intracerebral hemorrhage; IS, ischemic stroke; HTN, hypertension; CTRL, healthy control. Statistical comparisons for percentages were performed using chi-square test. Comparisons between means or medians were performed using a One-way ANOVA.

### RNA sequencing and data analysis

Blood samples from patients with spontaneous ICH within 48 hours of admission or acute IS patients were collected for transcriptome analysis. RNA isolation and sequencing were performed as previously described (19, 20). Library construction and sequencing were performed by Annoroad Gene Technology (Beijing, China) via Illumina’s NEBNext Ultra Directional RNA Library Prep Kit (NEB, Ipswich, USA). Clustering of the index-coded samples was performed on a cBot 2 cluster generation system via the TruSeq PE Cluster Kit v4-cBot-HS (Illumina, CA, USA) according to the manufacturer’s instructions. After cluster generation, the libraries were sequenced on an Illumina HiSeq 2500 platform for 150 bp paired-end reads. All reads were mapped to the human genome hg19 via the STAR2.4.1d aligner. The DESeq2 (22) and edgeR (23) packages were used to normalize the FPKM values and identify significant differentially expressed RNAs. Significantly differentially expressed genes (DEGs) between the two groups were identified as those with a |fold change| ≥ 2 and an adjusted p value (FDR) < 0.05. P values were corrected for multiple testing with the Benjamini–Hochberg method. Hierarchical clustering was performed, and heatmaps were generated on the basis of the normalized values of all DEGs using the R package. Venn diagrams and volcano plots were generated to visualize the consistently significant DEGs between the two cohorts. The RNA-seq data have been deposited into the Genome Sequence Archive in the National Genomics Data Center, China National Center for Bioinformation/Beijing Institute of Genomics, Chinese Academy of Sciences under accession number HRA001807, and they are publicly accessible at https://ngdc.cncb.ac.cn/gsa-human (19).

### DEG functional enrichment

Gene Ontology (GO) and Kyoto Encyclopedia of Genes and Genomes (KEGG) pathway analyses were performed to annotate the potential functions of the DEGs. A false discovery rate (FDR) < 0.05 was set as the cutoff for significantly enriched GO terms and KEGG pathways. Furthermore, gene set enrichment analysis (GSEA) was used to analyze the common DEGs in the two cohorts via the clusterProfiler R package, as previously described (24). The gene sets were analyzed on the basis of the KEGG and Reactome pathways. Enriched gene sets were assigned on the basis of a nominal p value<0.05 and a FDR <0.25.

### Construction of the lncRNA−mRNA coexpression network

To explore the correlation of differentially expressed lncRNAs and differentially expressed mRNAs, we constructed a lncRNA−mRNA coexpression network via Pearson correlation coefficient analysis. Coexpressed lncRNA−mRNA pairs were defined as those with a rho value > 0.85 and a p value <0.05. A correlation network was constructed via the OmicStudio tools at https://www.omicstudio.cn/tool.

### Weighted gene correlation network analysis

A co−expression network was constructed with the WGCNA package to identify the correlations among genes and identify highly correlated gene modules and potential biomarkers. The ICH, IS, HTN and CTRL group data were analyzed via the R package WGCNA (25). Pearson’s correlation analysis was performed to identify coexpressed genes, and an adjacency matrix was constructed on the basis of soft-thresholding (β = 9). Then, we created a topological overlap matrix (TOM) to visualize the connections among genes. Modules were identified via hierarchical clustering via the TOM and the dynamic tree cut algorithm. A gene significance > 0.2 and a module membership > 0.6 were calculated for individual modules to determine the most important genes. R > 0.5 and P < 0.05 were considered to indicate that a module was significant and should be selected for additional processing.

### Evaluation of immune cell composition via ImmuneCellAI

We evaluated the immune cell composition of the two comparison groups in the two cohorts via the ImmuneCellAI website (https://guolab.wchscu.cn/ImmuCellAI/), as previously described (26). The abundances of 24 immune cell types and 18 T-cell subtypes were calculated, and the abundance of immune cells in each sample was determined for further comparison. P<0.05 was considered to indicate statistical significance.

### Identification of candidate immune-related biomarkers with multiple machine learning algorithms

To identify immune-related biomarkers for ICH, we used least absolute shrinkage and selection operator (LASSO), support vector machine recursive feature elimination (SVM-RFE) algorithms, XGBoost-RET and Boruta algorithms to rank the importance of features according to RNA expression levels in all samples. The intersection of the candidate biomarkers was used to further assess their diagnostic value in discriminating ICH patients from patients in other groups according to the expression levels of RNAs via eight machine learning classification algorithms, namely, support vector machine (SVM), K-nearest neighbor (KNN), logistic regression (LR), random forest (RF), Gaussian naive Bayes (GNB), AdaBoost, light gradient boosting machine (LGBM) and eXtreme gradient boosting (XGB). The area under the curve (AUC), sensitivity, specificity, accuracy, positive predictive value (PPV), and negative predictive value (NPV) were computed. The programs used to run the algorithms and the specific protocols or tools used to assess diagnostic value on the Beckman Coulter DxAI platform (https://www.xsmartanalysis.com/beckman/login/).

### Validation via real-time polymerase chain reaction

Candidate biomarkers (CKAP4, BCL6 and TLR8) were selected for validation via quantitative real-time polymerase chain reaction (RT−PCR). Total RNA from peripheral blood white blood cells was isolated via TRIzol reagent (Invitrogen). cDNA synthesis was completed via the use of 1 µg of total RNA and a Transcriptor First Stand cDNA Synthesis Kit (Takara, Dalian, China). RT−PCR was performed via SYBR Master Mix (Yeasen, Shanghai, China) according to the manufacturer’s instructions. The RNA primers were designed via the NCBI Primer-BLAST website. The primers used in this study are listed in **Supplementary Table 9**. The target gene mRNA levels were quantified via normalization to those of the standard housekeeping gene gapdh, which served as an internal control.

### Cell culture

RAW264.7 cells were obtained from the Cell Resource Center, Peking Union Medical College (which is part of the National Science and Technology Infrastructure, the National Biomedical Cell-Line Resource, NSTI-BMCR; http://cellresource.cn). The cells were cultured in DMEM supplemented with 10% fetal bovine serum, 100 U/mL penicillin, and 100 mg/mL streptomycin in a 5% CO_2_ incubator at 37°C. Cells obtained from passages three to six were used in the study. For macrophage polarization, lipopolysaccharide (LPS, 100 ng/ml; Sigma−Aldrich; St. Louis, MO) and interferon-γ (IFN-γ, 20 ng/ml; PeproTech; Rocky Hill, NJ) were added to the culture mixture for 24 h to induce M1 polarization, and interleukin-4 (IL-4, 20 ng/ml; PeproTech; Rocky Hill, NJ) was added to the culture mixture for 24 h to induce M2 polarization.

### Western blot analysis

Cellular protein was extracted via RIPA lysis buffer containing a protease inhibitor cocktail (Roche). After homogenization on ice and centrifugation, total protein was mixed with loading buffer and separated on 4–12% NuPAGE Bis−Tris gels (Invitrogen) before being transferred to a nitrocellulose membrane. After being blocked with 5% nonfat milk containing Tris-buffered saline, the membranes were blotted with primary antibodies overnight at 4°C. The primary antibodies used were as follows: a monoclonal antibody against BCL6 (1:500, ab241549; Abcam, Cambridge, MA, USA), a polyclonal antibody against TLR8 (1:1000, ab8245; Abcam, Cambridge, MA, USA), a polyclonal antibody against CKAP4 (1:2000, 16686-1-AP; Proteintech, Wuhan, China), and a monoclonal antibody against GAPDH (1:10000, ab8245; Abcam, Cambridge, MA, USA). The membranes were subsequently washed four times for 5 min each with TBST buffer and incubated with a horseradish peroxide-conjugated secondary antibody (1:5000, SA00001-1, SA00001-2, Proteintech, Wuhan, China) at room temperature for 2 h. After washing, the membrane was developed with enhanced chemiluminescence reagent (Invitrogen, USA), and a ChemiDoc MP Imaging System (Bio-Rad, USA) was used for signal detection. Protein expression was quantified and normalized to that of GAPDH, which was used as an internal control. The signal density was quantified via ImageJ software (version 1.52a, NIH, USA).

### Histology and immunofluorescence

Spontaneous ICH mouse models were induced and assessed via MRI as described previously (27, 28). Animal experiments were approved by the Committee on the Ethics of Animal Experiments at Fuwai Hospital (approval No.: 0085-M-200-HX) and complied with the National Institutes of Health (NIH)’s Guide for the Care and Use of Laboratory Animals. The manuscript adheres to Animal Research: Reporting of *In Vivo* Experiments (ARRIVE) guidelines for reporting animal experiments. The brains were harvested and fixed in 4% paraformaldehyde for 6 h and then cryoprotected in 20% sucrose overnight at 4°C. Frozen coronal sections were cut at a thickness of 20 μm. Hematoxylin−eosin (HE) staining was applied to observe hemorrhages. For immunofluorescence staining, frozen brain sections were incubated in goat serum (ZSGB-BIO, Beijing, China) with 0.3% Triton X-100 for blocking. For BCL6, after above incubation, sections were blocked with Mouse on Mouse (MOM) Blocking Reagent (Vector Laboratories, MKB-2213-1, Burlingame, CA, USA) for one hour at room temperature to reduce non-specific binding, followed by incubation with primary antibodies overnight at 4°C. The primary antibodies used were as follows: mouse anti-BCL6 (1:100, ab241549; Abcam, Cambridge, MA, USA), rabbit anti-TLR8 (1:100, ab8245; Abcam, Cambridge, MA, USA), and rabbit anti-CKAP4 (1:100, 16686-1-AP; Proteintech, Wuhan, China). Rabbit or mouse isotype antibodies were used as negative controls. After several washes, the sections were incubated with secondary antibodies (Alexa Fluor 594-conjugated goat anti-mouse IgG, Alexa Fluor 488-conjugated goat anti-rabbit IgG; 1:300, ZSGB-BIO, Beijing, China) at room temperature for 30 min. Coverslips were mounted with Vecta Shield medium containing DAPI to stain the nuclei. All images were visualized via an FV3000 laser scanning confocal microscope.

### Statistical analysis

Statistical analysis was performed via SPSS 21.0 (IBM Corp., NY, USA). The sample distribution was determined via the Kolmogorov–Smirnov normality test. For parametric data, two-tailed unpaired Student’s t tests were used to evaluate differences between two groups. One-way ANOVA and the Bonferroni *post hoc* correction were performed when more than two groups were evaluated. The data are presented as the means ± standard deviations or medians (interquartile ranges). Statistical comparisons of percentages were performed via chi-square tests. For RNA sequencing analysis, DEGs were selected if significant differences (fold change ≥ 2 and FDR < 0.05) between two groups were observed via an unpaired Mann−Whitney test. Spearman’s correlation analysis was performed to investigate the correlations between ICH risk factors and candidate RNAs. Receiver operating characteristic (ROC) curve analysis and decision curve analysis (DCA) were used as accuracy indices for evaluating the diagnostic performance of the selected RNA panel. P < 0.05 was considered to indicate statistical significance.

## Results

### Characteristics and demographics of the study population

To investigate the expression profiles of mRNAs and lncRNAs associated with the occurrence and development of ICH, we performed RNA sequencing (RNA-seq) of the discovery and validation cohorts. The average age of the 233 subjects in this study was 57.58 ± 6.99 years (± SD), and 119 subjects (51.07%) were male. The demographics and characteristics of the ICH patients, IS patients, HTN controls and CTRLs in the discovery (n = 160) and validation (n = 73) cohorts are shown in **Tables 1**, **2**. No significant differences in sex, age or incidence rates of diabetes mellitus or hyperlipidemia existed among the patients with ICH, IS, or HTN and the matched controls in either the discovery or validation cohorts. The workflow of this study is shown in **Supplementary Figure 1**.

### LncRNA and mRNA expression profiles are significantly altered in ICH patients in both the discovery and validation cohorts

**Figure 1:**
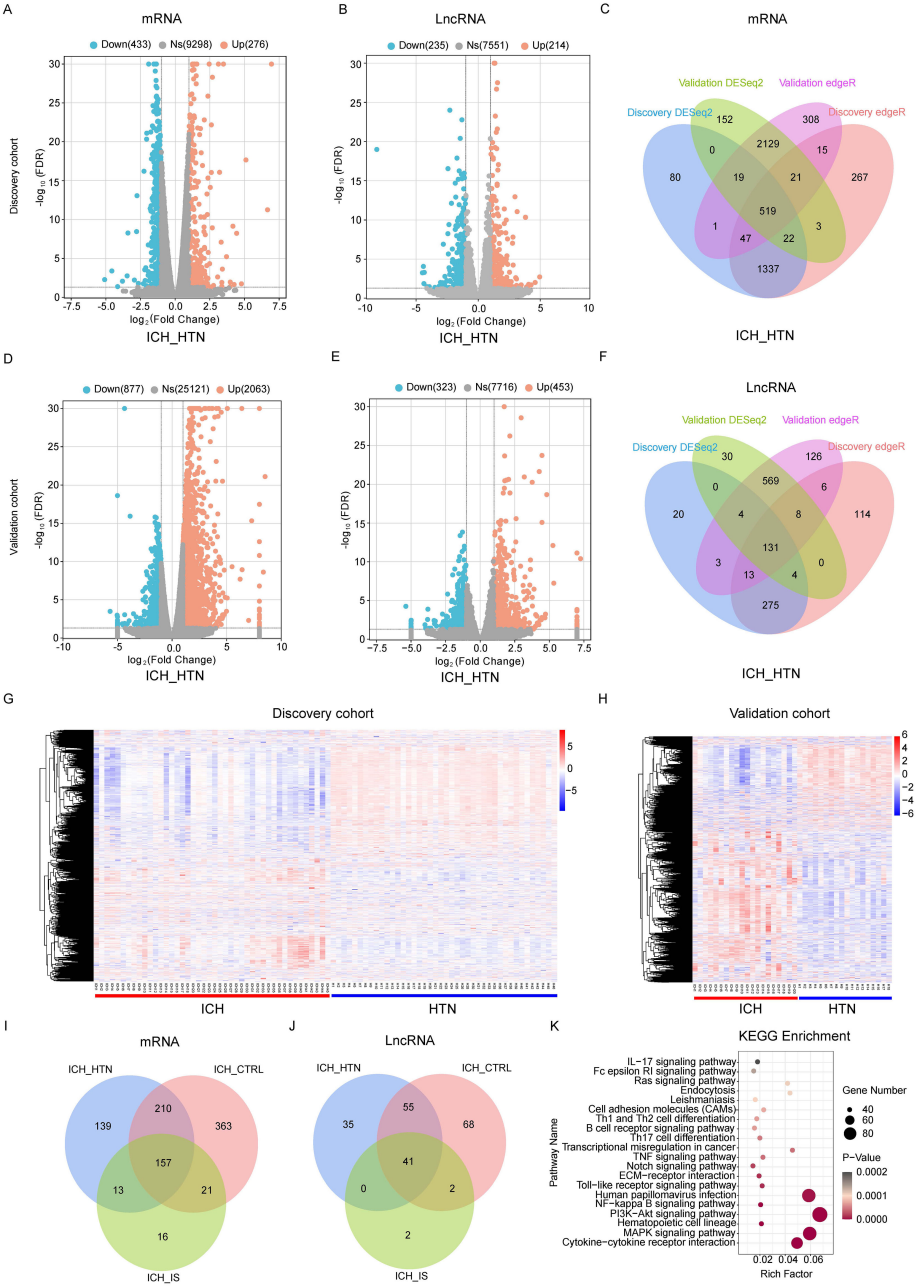
Differentially expressed genes between intracerebral hemorrhage (ICH) patients and hypertension (HTN) controls in the discovery and validation cohorts. Volcano plots of the mRNA **(A, D)** and lncRNA **(B, E)** expression profiles of ICH patients and HTN controls (fold change ≥2 and FDR < 0.05) in the discovery **(A, B)** and validation **(D, E)** cohorts. The red dots represent upregulated genes, and the blue dots represent downregulated genes. **(C, F)** Venn diagram showing the consistently altered mRNAs **(C)** and lncRNAs **(F)** in ICH patients compared with HTN controls in the discovery and validation cohorts according to both the DESeq2 and edgeR methods. **(G, H)** Hierarchical clustering of genes that were consistently differentially expressed between ICH patients and HTN controls in the discovery **(G)** and validation **(H)** cohorts. Blue represents downregulated genes, red represents upregulated genes, and gray represents genes whose expression did not change. Each column represents a sample, and each row represents a single gene. **(I, J)** Venn diagram showing the consistently differentially expressed mRNAs **(I)** and lncRNAs **(J)** between the comparison groups (ICH vs. HTN, ICH vs. CTRL and ICH vs. IS) in both cohorts. **(K)** The top 20 Kyoto Encyclopedia of Genes and Genomes (KEGG) pathways associated with consistently differentially expressed mRNAs. Statistical significance levels were corrected for multiple testing using the Benjamini–Hochberg procedure.

In total, 519 mRNAs and 131 lncRNAs (fold change > 2 and FDR < 0.05) were consistently significantly differentially expressed between ICH patients and HTN controls (**Figures 1A-F**; **Supplementary Tables 1, 2**) in both cohorts according to the DESeq2 and EdgeR results. Volcano plots were generated to evaluate the variation and reproducibility of lncRNA and mRNA expression in ICH patients and HTN controls in the discovery (**Figures 1A, B**) and validation (**Figures 1D, E**) cohorts. The expression patterns of mRNAs and lncRNAs in ICH patients and HTN controls in the discovery (**Figure 1G**) and validation (**Figure 1H**) cohorts were distinguished via hierarchical clustering and heatmaps. Similarly, 751 mRNAs and 166 lncRNAs were consistently significantly differentially expressed between ICH patients and CTRLs (**Supplementary Figures 2A, B**; **Supplementary Tables 3, 4**), and 207 mRNAs and 45 lncRNAs were consistently significantly differentially expressed between ICH patients and IS patients according to the same methods (**Supplementary Figures 2C, D**; **Supplementary Tables 5, 6**). Moreover, we identified 157 differentially expressed mRNAs and 41 differentially expressed lncRNAs that overlapped among the three comparison groups (ICH vs. HTN, ICH vs. CTRL and ICH vs. IS) and selected them for further analysis (**Figures 1I, J**).

### Construction of the lncRNA−mRNA coexpression network

Genes with the same expression pattern may function together. To explore the relationships between the 157 differentially expressed mRNAs and 41 differentially expressed lncRNAs in all the samples, we constructed a lncRNA−mRNA coexpression network via Pearson correlation coefficient analysis. We defined lncRNA−mRNA pairs as coexpressed if the absolute value of rho was >0.85 and the p value was < 0.05. The lncRNA−mRNA coexpression network contained 49 nodes (36 mRNAs and 13 lncRNAs) and 52 connections. The top 5 nodes were RP11-574K11.5, CTB-61M7.2, RP11-483F11.7, AC098823.3 and LINC00671, with a degree >5 (**Supplementary Figure 3**). The top 10 lncRNA−mRNA coexpression pairs were RP11−574K11.5− S100A12, RP11−574K11.5− GPR84, RP11−483F11.7− ENTPD7, RP11−36B6.1− AP3B2, LINC00671− MMP9, RP11−574K11.5− HP, RP11−483F11.7− UGCG, CTB−61M7.2− SLC2A3, RP11−574K11.5− C19orf59, and RP11−574K11.5− SLC51A (**Supplementary Table 7**), which may play vital roles in the regulation of ICH pathogenesis.

### Functional enrichment and pathway analysis of DEGs

**Figure 2:**
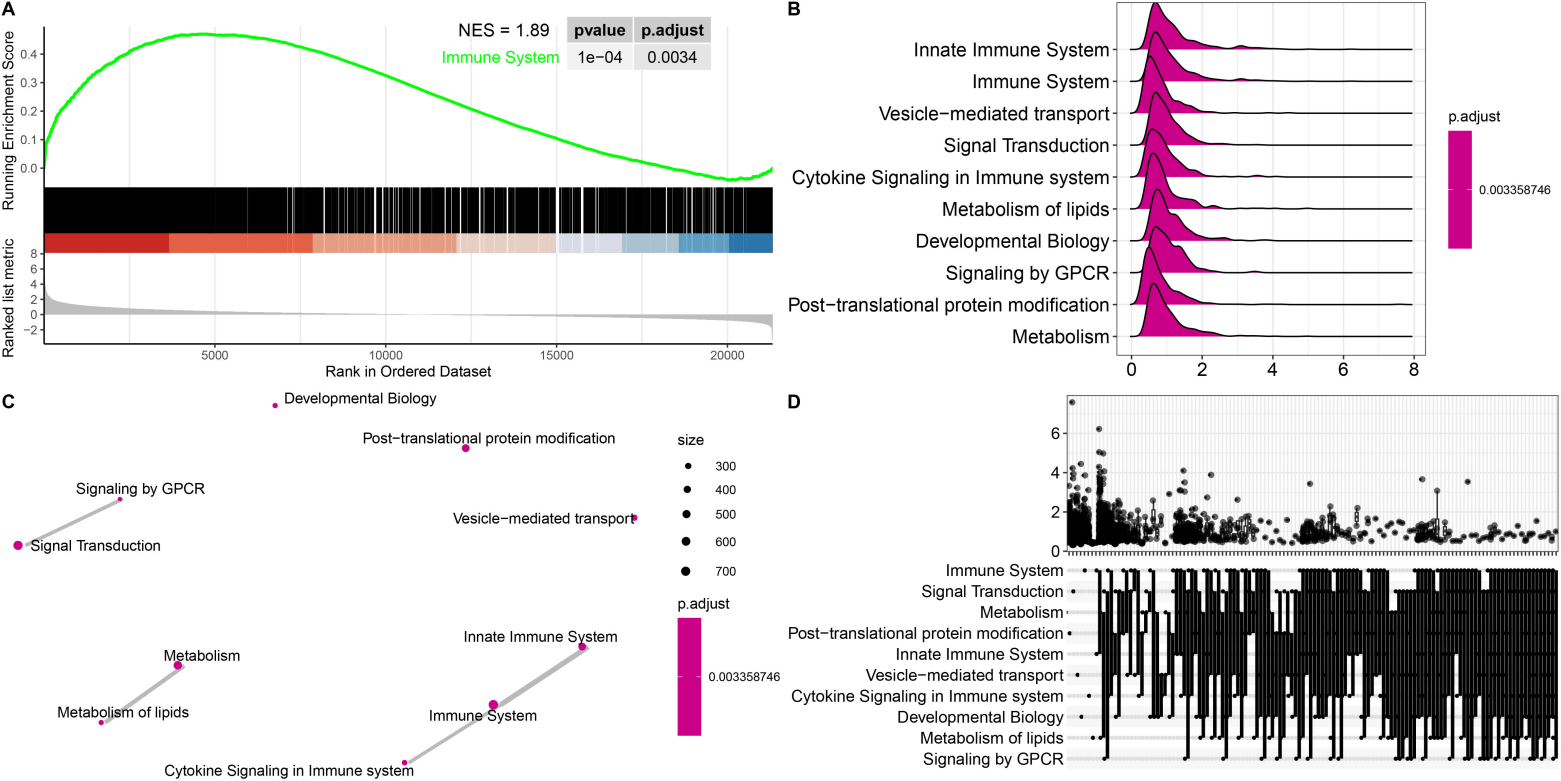
Gene set enrichment analysis (GSEA) of representative pathways associated with genes significantly differentially expressed in ICH patients. **(A)** Enrichment plot showing the immune system pathway with the highest enrichment score. **(B-D)** Ridge plot **(B)**, network diagram **(C)** and UpSet plot **(D)** showing the top 10 pathways in the annotated gene sets. Statistical significance levels were corrected for multiple testing using the Benjamini–Hochberg procedure.

Functional enrichment analyses were performed to further explore the functions of the DEGs between patients with ICH and matched controls. We observed that the enriched GO terms for the significant DEGs included immune response, immune system process, regulation of biological response, and receptor binding (**Supplementary Figure 4**), which are closely related to the pathophysiology of ICH. KEGG pathway analysis of the DEGs revealed that the cytokine−cytokine receptor interaction, MAPK signaling pathway, PI3K–Akt signaling pathway, ECM–receptor interaction, Notch signaling pathway, B-cell receptor signaling pathway, Th1 and Th2 cell differentiation, TNF signaling pathway and Th17 cell differentiation pathways were significantly enriched in both cohorts (**Figure 1K**). Gene set enrichment analysis (GSEA) was performed to identify the signaling pathways involved. Reactome enrichment revealed that the top pathways were the immune system (NES=1.89, P= 0.0034) (**Figure 2A**), metabolism of lipids, B-cell receptor signaling pathways, HIF-1 signaling pathways, Fc gamma R-mediated phagocytosis, osteoclast differentiation, and Th1 and Th2 cell differentiation (**Figures 2B-D**; **Supplementary Figures 5A-D**). These results demonstrated that the immune response may play important roles in the pathogenesis of ICH.

### Immune cell abundance is significantly altered in ICH patients according to immune cell infiltration analysis

**Figure 3:**
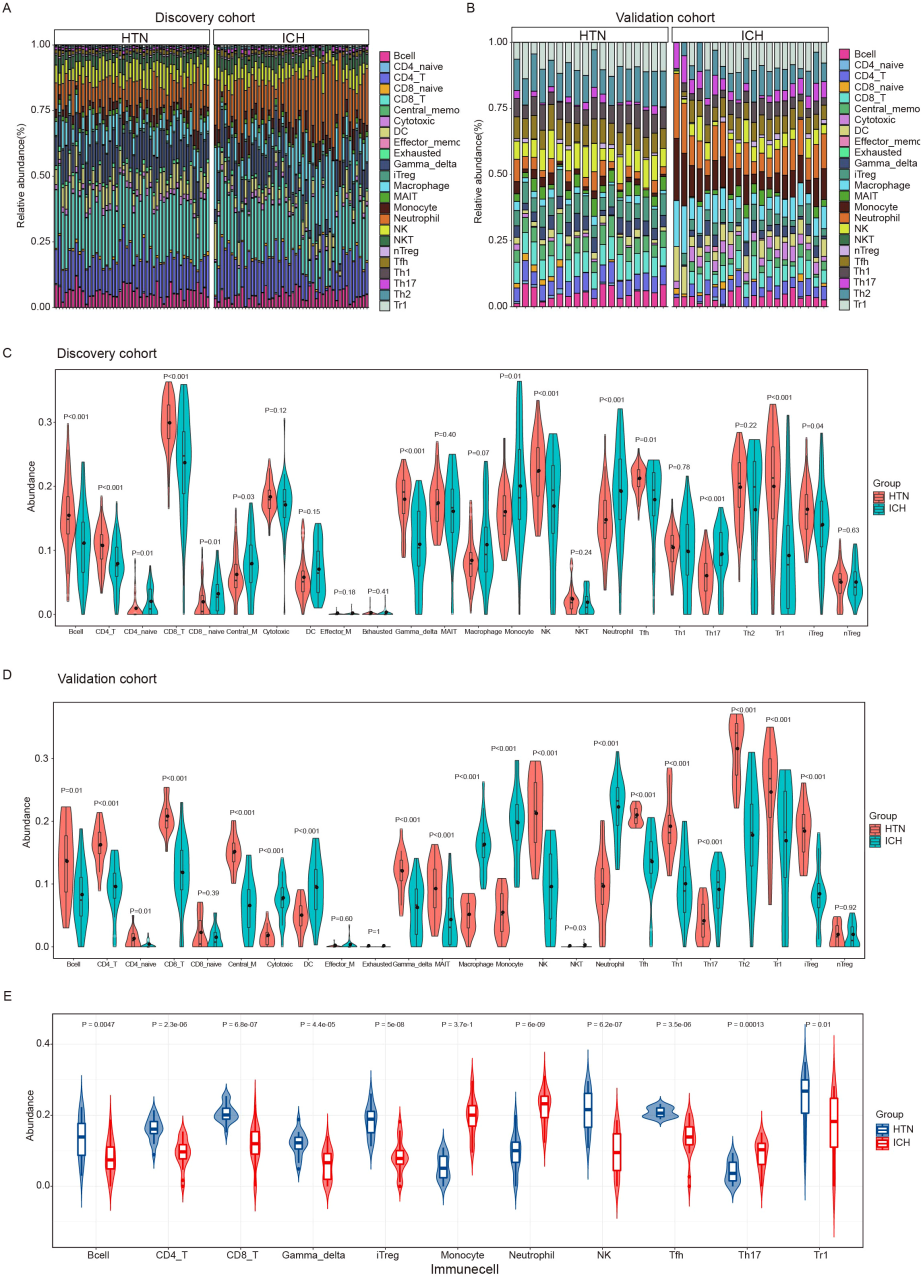
Immune cell infiltration analysis of the intracerebral hemorrhage (ICH) and hypertension (HTN) control groups by ImmuneCellAI. **(A, B)** Stacked bar plots showing the relative proportions of 24 immune cell subsets in the discovery **(A)** and validation **(B)** cohorts. **(C, D)** Violin diagrams showing the differences between ICH patients and HTN controls for 24 infiltrating immune cell types in the discovery **(C)** and validation **(D)** cohorts. **(E)** Violin diagrams showing 11 consistent differences in infiltrating immune cell types between ICH patients and HTN controls in both cohorts. The data were assessed using the Benjamini−Hochberg (BH) method.

To explore the immune cell composition after ICH, we used ImmuneCellAI to estimate the abundances of 24 immune cell types in the two cohorts. The abundances of 24 immune cells in each sample in the discovery and validation cohort are shown in **Figures 3A, B**. Comparisons of the proportions of 24 immune cell types between ICH patients and HTN controls in the discovery and validation cohorts are shown in **Figures 3C, D**. Overall, 11 immune cell types overlapped between the two cohorts, as shown in the boxplot in **Figure 3E**. The results revealed that the numbers of CD4 T cells, CD8 T cells, type 1 regulatory T (Tr1) cells, induced regulatory T (iTreg) cells, follicular T-helper (Tfh) cells, gamma-delta T cells, Th17 cells, and other immune cells, including B cells, monocytes, natural killer (NK) cells and neutrophils, significantly differed between ICH patients and HTN controls (P<0.05). Moreover, monocyte, neutrophil and Th17 cell numbers increased, but the numbers of other cell types decreased significantly after ICH. These results indicate that the proportion and distribution of immune cells are altered in ICH patients and may play crucial roles in the pathogenesis of ICH.

### Identification of immune-related DEGs via weighted gene coexpression network analysis and GSEA

**Figure 4:**
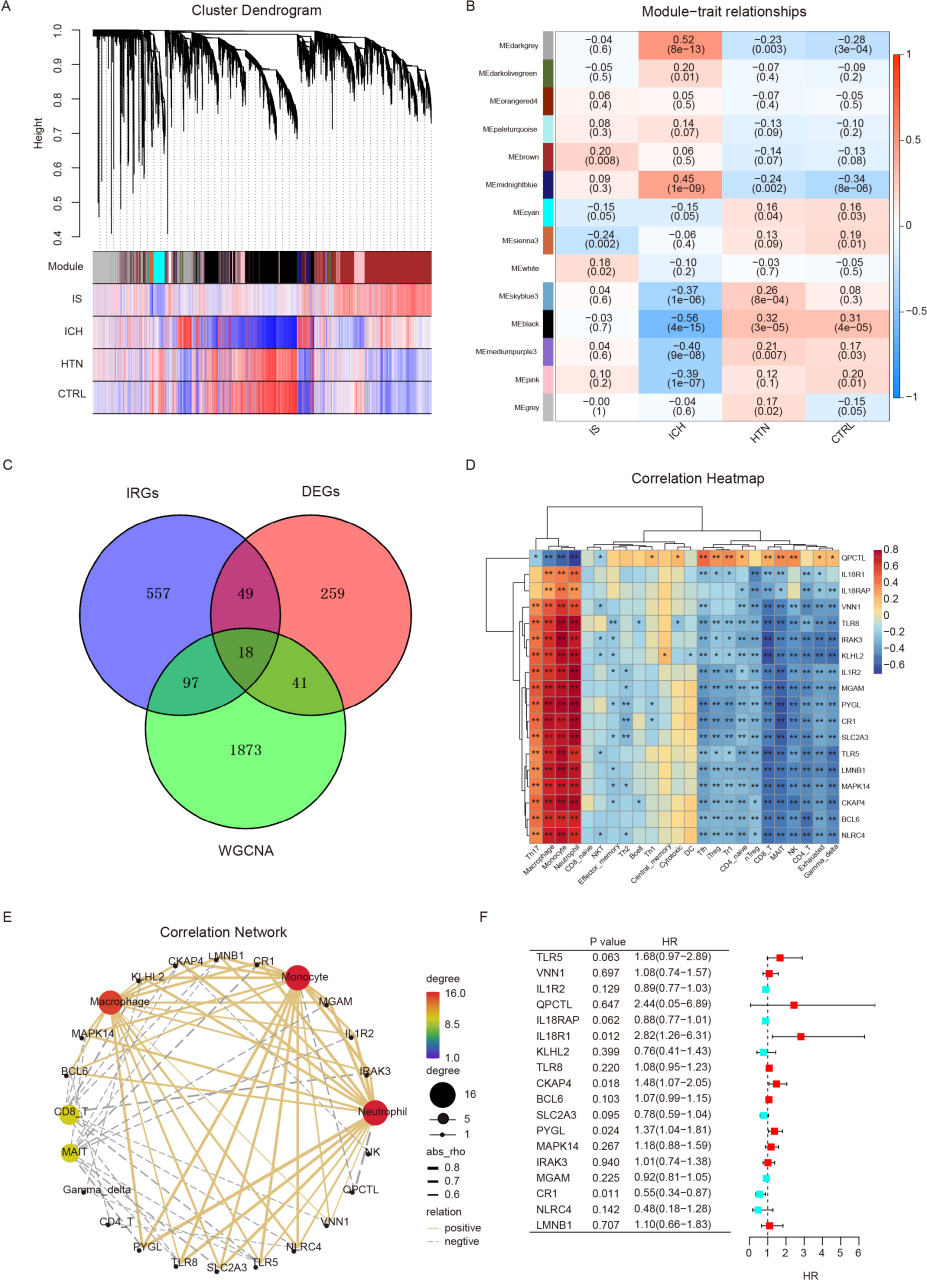
Differentially expressed immune-related genes were screened via weighted gene coexpression network analysis (WGCNA) and GSEA. **(A)** Cluster dendrogram showing the gene modules among the four groups. Genes were divided into various modules by hierarchical clustering, and different colors represent different modules. **(B)** Heatmap of module-trait relationships. The black and dark gray modules were significantly associated with intracerebral hemorrhage (ICH) (R>0.5, p < 0.001). **(C)** Venn diagram showing eighteen differentially expressed immune-related genes (IRGs) identified via WGCNA and immune system genes (GSEA). **(D, E)** Heatmap **(D)** and correlation network **(E)** showing the relationships between eighteen IRGs and immune cell infiltration. **(F)** Univariate Cox regression analysis of eighteen IRGs for prognostic assessment. *p<0.05, **p<0.01.

To identify the specific modules associated with ICH, we performed WGCNA with the R package and performed network and module detection. Then, gene cluster dendrograms were constructed, and dynamic tree cutting was performed (**Figure 4A**). Fourteen modules of coexpressed genes were identified, and the correlations among different modules and differential groups were assessed to determine the most significant correlations. Notably, the dark gray (R = 0.52, P =8e-13) and black (R = -0.56, P =4e-15) modules were significantly different between the ICH group and the other groups on the basis of the criteria of absolute correlation > 0.5 and p value < 0.05 (**Figure 4B**). A total of 2029 key module genes related to ICH were identified. According to previous GSEA and immune cell infiltration analysis results, the immune system plays a vital role in the pathogenesis of ICH; subsequently, we identified 18 intersecting genes by overlapping 2029 key module genes related to ICH, 721 key genes related to the immune system and 367 DEGs (ICH vs. HTN and ICH vs. CTRL); these genes were identified as immune-related DEGs in ICH (**Figure 4C**). Furthermore, we analyzed the correlations between the expression levels of eighteen immune-related DEGs and immune cell infiltration in ICH patients via Spearman’s analysis. The results revealed that the expression levels of most immune-related DEGs were positively correlated with the abundance of Th17 cells, macrophages, monocytes and neutrophils and negatively correlated with Tfh cells, iTreg cells, Tr1 cells, CD4-naive cells, nTreg cells, CD8 T cells, MAIT cells, NK cells, CD4-T cells, and exhausted and gamma delta T cells; however, QPCTL exhibited the opposite pattern (P<0.05) (**Figures 4D, E**). Additionally, univariate Cox regression was performed to evaluate these eighteen immune-related DEGs as predictors of ICH prognosis, and the results revealed that the expression levels of IL18R1 (HR=2.82, 95% CI: 1.26–6.31, P=0.012), CKAP4 (HR=1.48, 95% CI: 1.07–2.05, P=0.018), PYGL (HR=1.37, 95% CI: 1.04–1.81, P=0.024), and CR1 (HR=0.55, 95% CI: 0.34–0.87, P=0.011) were significantly associated with the prognosis of ICH patients (**Figure 4F**).

### Identification of immune-related diagnostic biomarkers for ICH with multiple classification algorithms

**Figure 5:**
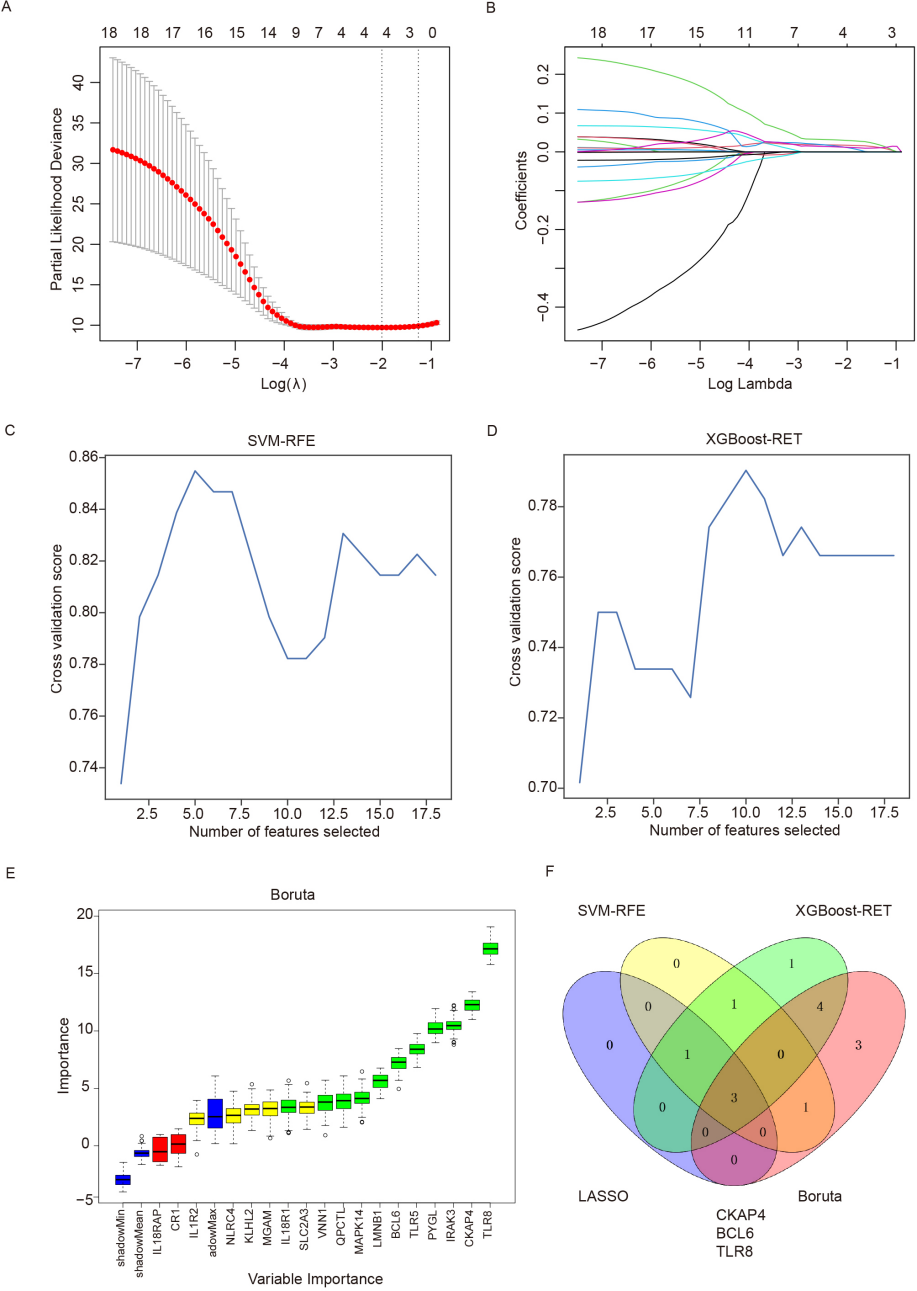
Detection of immune-related diagnostic markers via four classification algorithms. **(A)** LASSO regression algorithm for screening diagnostic markers. The relationship between the binomial deviation and log(λ) was determined via 10-fold cross-validation (CV). **(B)** The coefficients of 18 feature genes are shown as log(λ). Different colors represent different genes. **(C)** SVM-RFE was used to screen biomarkers. **(D)** The XGBoost-RET algorithm was used to screen biomarkers. **(E)** The Boruta algorithm was used to screen biomarkers. **(F)** Venn diagram showing the intersection of the diagnostic markers obtained with the four algorithms. SVM-RFE: support vector machine recursive feature elimination, LASSO: least absolute shrinkage and selection operator, XGBoost: extreme gradient boosting.

To further evaluate the eighteen immune-related DEGs as biomarkers for the diagnosis of ICH, we used the LASSO, SVM-RFE, XGBoost-RET and Boruta algorithms to rank the importance of the features according to their expression levels in all the samples. We reduced the number of dimensions through LASSO regression and selected 4 genes to construct a diagnostic model for ICH (**Figures 5A, B**). Similarly, we identified 5 genes from SVM-RFE and 10 genes from XGBoost-RET with the highest scores as optimal diagnostic tools for ICH (**Figures 5C, D**). Furthermore, we carried out feature selection via the Boruta algorithm and identified 11 genes as important, of which TLR8, CKAP4 and BCL6 were consistent with the top discriminatory biomarkers identified by the above three models (**Figure 5E**). Ultimately, 3 candidate genes (CKAP4, BCL6 and TLR8) were identified by intersection, and their diagnostic value was further assessed (**Figure 5F**).

### Validation of candidate biomarkers via quantitative real-time polymerase chain reaction

**Figure 6:**
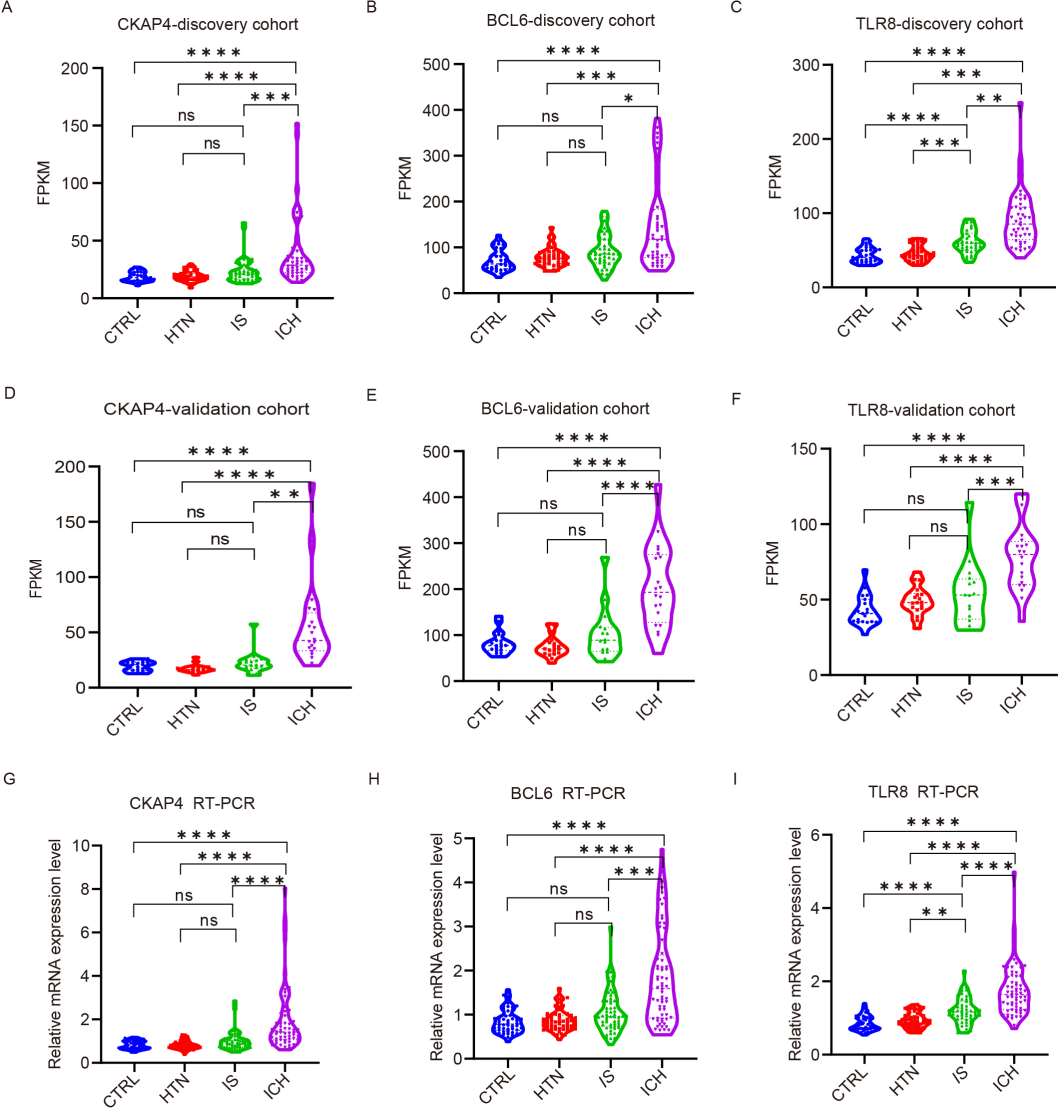
Validation of candidate gene expression levels via quantitative real-time polymerase chain reaction (RT−PCR). **(A-F)** The expression levels of three candidate genes in the discovery **(A-C)** and validation **(D-F)** cohorts among the four groups according to FPKM values. CKAP4 **(A, D)**, BCL6 **(B, E)** and TLR8 **(C, F)**. **(G-I)** RT−PCR results validated the expression levels of CKAP4 **(G)**, BCL6 **(H)** and TLR8 **(I)** among 64 intracerebral hemorrhage (ICH) patients, 59 ischemic stroke (IS) patients, 60 hypertension (HTN) controls and 50 healthy controls (CTRLs). The data are presented as the median (interquartile range). *p<0.05, ***p<0.001, **p<0.01, ****p<0.0001. ns, no significance. Statistical significance was assessed using one-way ANOVA.

To explore the ability of the three candidate genes as potential biomarkers to distinguish between ICH patients and other groups, we detected the expression levels of CKAP4, BCL6 and TLR8 in ICH patients, IS patients, HTN controls and CTRLs as FPKM values in both cohorts. The results demonstrated that CKAP4, BCL6 and TLR8 levels were significantly upregulated in ICH patients compared with those in IS patients, HTN controls and CTRLs but were not significantly different between IS patients and HTN controls (or CTRLs) in both the discovery (**Figures 6A-C**) and validation cohorts (**Figures 6D-F**). These three candidate biomarker genes were subsequently validated via RT−PCR in technical replicates of the four groups, and the results were consistent with those obtained via RNA sequencing (**Figures 6G−I**). These results indicate that CKAP4, BCL6 and TLR8 are specifically upregulated in patients with ICH and could be used as diagnostic biomarkers for ICH.

### Diagnostic value of the three candidate biomarkers for ICH patients

Given that the above three genes were significantly differentially expressed according to both RNA sequencing and RT−PCR, we determined their diagnostic value with eight machine learning classification algorithms. The average performance values of the three candidate biomarkers for ICH based on accuracy and the area under the curve (AUC) in the training set, validation set and test set. Among these classifiers, the performance of GNB was superior to that of the other seven algorithms; the AUC for the model was 0.93 in the training set, 0.91 in the validation set, and 0.97 in the test set (**Table 3**). These results indicate that these three candidate biomarkers have great clinical value in the diagnosis of ICH.

**Table 3:** Classification performance for the three-candidate RNA signatures in ICH patients.

Classifier	Set	AUC	Accuracy	Sensitivity	Specificity	PPV	NPV
LR	Training set	0.94	0.88	0.83	0.95	0.94	0.84
	Validation set	0.92	0.84	0.84	0.96	0.90	0.81
	Test set	0.86	0.79	0.85	0.83	0.91	0.63
XGB	Training set	1.00	0.98	0.99	1.00	1.00	0.97
	Validation set	0.91	0.85	0.84	0.95	0.87	0.87
	Test set	0.86	0.84	0.92	0.83	0.86	0.80
LGBM	Training set	0.98	0.95	0.96	0.96	0.96	0.94
	Validation set	0.91	0.84	0.86	0.91	0.87	0.83
	Test set	0.90	0.74	0.69	1.00	0.83	0.57
RF	Training set	1.00	0.98	1.00	1.00	1.00	0.96
	Validation set	0.93	0.87	0.84	0.93	0.92	0.84
	Test set	0.89	0.74	1.00	0.67	0.90	0.56
AdaBoost	Training set	1.00	0.99	1.00	1.00	1.00	0.97
	Validation set	0.94	0.85	0.94	0.89	0.91	0.82
	Test set	0.76	0.68	0.69	1.00	0.82	0.50
GNB	Training set	0.93	0.87	0.85	0.91	0.90	0.85
	Validation set	0.91	0.83	0.90	0.91	0.83	0.84
	Test set	0.97	0.90	0.85	1.00	0.92	0.83
SVM	Training set	0.93	0.85	0.86	0.87	0.87	0.85
	Validation set	0.94	0.82	0.94	0.89	0.82	0.85
	Test set	0.96	0.84	0.85	1.00	0.81	1.00
KNN	Training set	0.96	0.86	0.86	0.91	0.96	0.80
	Validation set	0.90	0.79	0.84	0.89	0.87	0.76
	Test set	0.92	0.63	0.62	1.00	1.00	0.00

LR, logistic regression; XGB, eXtreme Gradient Boosting; LGBM, Light Gradient Boosting machine; GNB, Gaussian naive Bayes; RF, Random Forest; SVM, support vector machines; KNN, k-Nearest Neighbor; PPV, positive predictive value; NPV, negative predictive value; AUC, area under curve.

**Figure 7:**
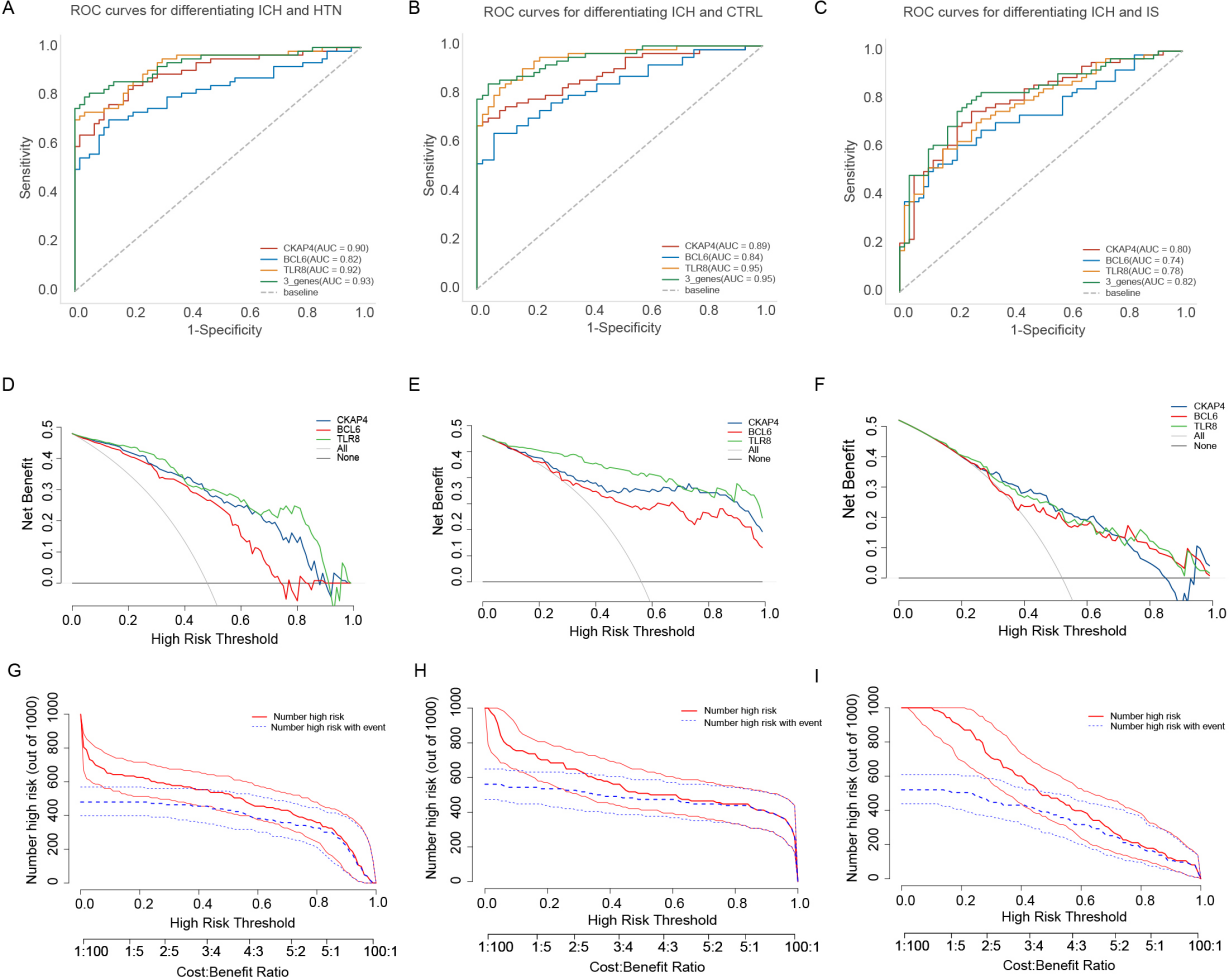
Diagnostic value of the candidate genes in intracerebral hemorrhage (ICH) patients. **(A)** Receiver operating characteristic (ROC) curves were generated using the expression levels of CKAP4, BCL6 and TLR8 individually or in combination to differentiate patients with ICH from hypertension controls (n = 64 vs. 60). **(B)** ROC curves were generated using the expression levels of CKAP4, BCL6 and TLR8 to differentiate patients with ICH from healthy controls in all samples (n = 64 vs. 50). **(C)** ROC curves of CKAP4, BCL6 and TLR8 were generated to differentiate ICH patients from IS patients in all samples (n = 64 vs. 59). **(D-F)** DCA curves of CKAP4, BCL6 and TLR8 were generated to differentiate ICH patients from HTN controls **(D)**, CTRLs **(E)** and IS patients **(F)** in all samples. **(G-I)** Clinical impact curves of the combination of CKAP4, BCL6 and TLR8 for discriminating ICH patients from HTN controls **(G)**, CTRLs **(H)** and IS patients **(I)**.

Receiver operating characteristic (ROC) curve analysis was subsequently performed to explore the potential diagnostic value of the three candidate biomarkers for ICH. The AUCs of the CKAP4, BCL6 and TLR8 signatures for differentiating patients with ICH from HTN controls were 0.90, 0.82, and 0.92, respectively (**Figure 7A**). The AUC values of the CKAP4, BCL6 and TLR8 signatures for differentiating patients with ICH and CTRLs were 0.89, 0.84 and 0.95, respectively (**Figure 7B**); the AUC values of the CKAP4, BCL6 and TLR8 signatures for differentiating patients with ICH and IS patients were 0.80, 0.74 and 0.78, respectively (**Figure 7C**). The combination of CKAP4, BCL6 and TLR8 for differentiating patients with ICH from HTN controls, CTRLs and IS patients had AUC values of 0.93 (95% CI: 0.89–0.98), 0.95 (95% CI: 0.92–0.99) and 0.82 (95% CI: 0.74–0.89), respectively, with sensitivities of 81.3%, 84.4% and 75%, respectively, and specificities of 100%, 96% and 79.7%, respectively (**Figures 7A−C**). We used DCA to evaluate the clinical utility of the three candidates by qualifying the net benefit at a distinct threshold. The curve shows that the number of positive cases predicted by the model was close to the actual number of positive cases. As the risk threshold increased, the number of cases predicted by the model approached the actual number of cases. As expected, the DCA results revealed that CKAP4, BCL6 and TLR8 had similar clinical values in the diagnosis of ICH when these patients were differentiated from CTRLs and IS patients. Compared with CKAP4 and BCL6, TLR8 had greater clinical value (**Figures 7D-F**). On the basis of the DCA results, we further plotted clinical impact curves to evaluate the clinical utility of the diagnostic model. The clinical impact curves of the combination of the three biomarkers showed that the predicted probability coincided well with the actual probability of differentiating patients with ICH from HTN controls, CTRLs and IS patients (**Figures 7G-I**), suggesting that the diagnostic model had excellent predictive value. These results indicate that CKAP4, BCL6 and TLR8 are diagnostic biomarkers for ICH either individually or in combination.

### Correlation analysis of three candidate biomarkers and clinical characteristics

To further assess the associations of the three candidate genes with the clinical characteristics of ICH patients, we performed Spearman’s correlation analysis to evaluate the correlations between the expression levels of CKAP4, BCL6 and TLR8 and the clinical characteristics of ICH patients. CKAP4, BCL6 and TLR8 expression levels were positively correlated with white blood cell counts and glucose levels and negatively correlated with low-density lipoprotein cholesterol (LDL-C), triacylglycerol (TG), total cholesterol (TC), uric acid (UA) and apolipoprotein A (ApoA) levels in ICH patients (P < 0.05). TLR8 and CKAP4 were positively correlated with direct bilirubin (DBIL) levels and negatively correlated with sex and HDL-C and apolipoprotein B (ApoB) levels (P < 0.05). BCL6 expression levels were positively correlated with SBP and DBP and negatively correlated with red blood cell (RBC) counts and hemoglobin (Hb) levels (P < 0.05) (**Supplementary Figure 6**; **Table 4**). These results indicate that CKAP4, BCL6 and TLR8 may be involved in the pathogenesis of ICH.

**Table 4:** Correlation between three RNA levels and baseline characteristic in ICH patients.

Parameters	TLR8		CKAP4		BCL6	
	Coefficient	*P-value*	Coefficient	*P-value*	Coefficient	*P-value*
Age, y	-0.08	0.344	-0.02	0.762	0.04	0.634
Sex (men)	-0.19*	0.015	-0.20*	0.013	-0.08	0.290
BMI, kg/m^2^	0.02	0.848	-0.11	0.152	0.03	0.679
SBP, mmHg	0.12	0.129	0.12	0.123	0.19*	0.016
DBP, mmHg	0.15	0.060	0.13	0.112	0.17*	0.033
WBC, 10^9^/L	0.44***	<0.001	0.34***	<0.001	0.48***	<0.001
RBC, 10^12^/L	-0.14	0.089	-0.15	0.059	-0.21**	0.008
Hb, g/L	-0.13	0.116	-0.13	0.100	-0.24**	0.003
PLT, 10^9^/L	-0.10	0.208	-0.16*	0.045	-0.06	0.447
HDL-C, mmol/L	-0.25**	0.001	-0.19*	0.015	-0.13	0.094
LDL-C, mmol/L	-0.30***	<0.001	-0.31***	<0.001	-0.17*	0.035
TC, mmol/L	-0.40***	<0.001	-0.37***	<0.001	-0.24***	<0.001
TG, mmol/L	-0.19*	0.016	-0.23**	0.004	-0.18*	0.024
Glucose, mmol/L	0.19*	0.018	0.17*	0.035	0.25**	0.001
UA, μmol/L	-0.18*	0.023	-0.20*	0.012	-0.31***	<0.001
TBIL, μmol/L	0.09	0.244	0.07	0.378	-0.04	0.664
DBIL, μmol/L	0.31***	<0.001	0.26***	0.001	0.12	0.131
BUN, mmol/L	0.08	0.296	0.04	0.660	0.06	0.492
ApoA, g/L	-0.31***	<0.001	-0.24**	0.002	-0.20*	0.014
ApoB, g/L	-0.25**	0.002	-0.25**	0.002	-0.06	0.464
Smoking	0.01	0.938	0.06	0.470	0.00	0.980
Drinking	0.13	0.102	0.09	0.242	0.03	0.707

ICH, Intracerebral hemorrhage; BMI, Body mass index; SBP, Systolic blood pressure; DBP, Diastolic blood pressure; TC, Total cholesterol; TG, Triacylglycerol; HDL-C, High-density lipoprotein cholesterol; LDL-C, Low-density lipoprotein cholesterol; GLU, Glucose; UA, Uric acid; TBIL, Total bilirubin; BUN, Blood urea nitrogen; WBC, White blood cell; RBC, Red blood cell; Hb, hemoglobin; DBIL, direct bilirubin; ApoA, apolipoprotein A; ApoB, apolipoprotein B; PLT, platelet. *P < 0.05; **P<0.01; ***P<0.001.

### External and experimental validation of the expression levels of three candidate immune-related biomarkers of ICH

**Figure 8:**
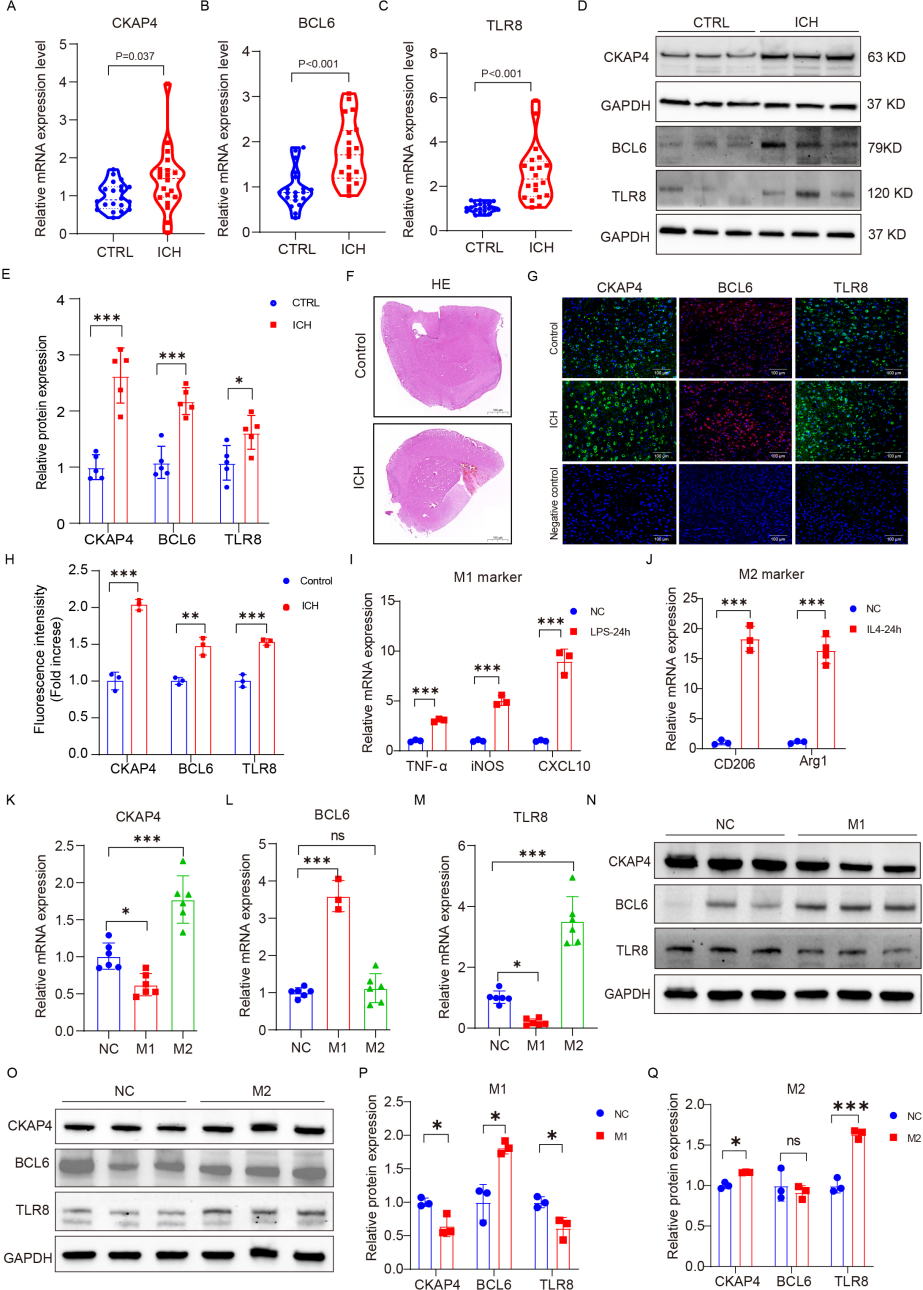
External and experimental validation. Comparison of CKAP4 **(A)**, BCL6 **(B)** and TLR8 **(C)** expression between ICH patients and CTRLs in an independent cohort (n=20 per group). **(D, E)** Western blot analysis of the expression levels of CKAP4, BCL6 and TLR8 in ICH patients and CTRLs in peripheral blood mononuclear cells (n=5 per group). **(F)** Representative images of HE staining in the ICH mice brain tissue and control groups. Scale bars, 100 µm. **(G, H)** Representative images of brain sections showing CKAP4, BCL6 and TLR8 staining in ICH mice and the control group; DAPI is shown in blue; BCL6 is shown in red; CKAP4 and TLR8 are shown in green. Scale bars, 100 µm. **(I, J)** The expression levels of M1 (TNF-α, iNOS, and CXCL10) and M2 (Arginase 1 and CD206) marker genes in RAW264.7 cells after stimulation with LPS (100 ng/ml), INF-γ (20 ng/ml) or IL4 (20 ng/ml) for 24 hours (n=3 per group). **(K−M)** The relative mRNA expression levels of CKAP4 **(K)**, BCL6 **(L)** and TLR8 **(M)** in M1 and M2 macrophages (n=3-6 per group). **(N-O)** Western blot analysis of the expression levels of CKAP4, BCL6 and TLR8 in M1 and M2 macrophages. **(P, Q)** Relative CKAP4, BCL6 and TLR8 protein expression levels in M1 and M2 macrophages (n=3 per group). The data are presented as the means ± SDs. *p<0.05; **p<0.01, ***p<0.001. ns, no significance. Statistical significance was assessed using 2-tailed Student’s *t* test **(A−E, H-J, P−Q)** or one-way ANOVA **(K−M)**.

To further verify the clinical utility of these three candidate biomarkers for the diagnosis of patients with ICH, we recruited 20 patients with ICH and 20 CTRLs as another independent cohort for validation. CKAP4, BCL6 and TLR8 mRNA (**Figures 8A-C**) and protein (**Figures 8D, E**) levels were significantly higher in patients with ICH than in those with CTRLs (**Supplementary Table 8**). We subsequently detected the expression levels of CKAP4, BCL6 and TLR8 in ICH model mice and found that CKAP4, BCL6 and TLR8 were elevated in brain tissues after hemorrhage (**Figures 8F-H**), which was consistent with the RNA sequencing and RT−PCR results. Additionally, we further investigated the functional roles of these three genes in macrophages. RAW264.7 cells were polarized to the M1 or M2 macrophage phenotype via treatment with LPS plus IFN-γ or IL-4, respectively. M1 macrophages expressed the M1 markers TNFα, iNOS and CXCL10 (**Figure 8I**); in contrast, M2 macrophages expressed the M2 markers arginase 1 (Arg1) and CD206 (**Figure 8J**), suggesting that RAW264.7 cells were successfully polarized to M1 and M2 macrophages. We found that CKAP4 and TLR8 levels were decreased in M1 macrophages and increased in M2 macrophages, whereas BCL6 expression was increased in M1 macrophages but not in M2 macrophages at both the mRNA (**Figures 8K−M**) and protein levels (**Figures 8N−Q**). These results indicate that BCL6 may promote M1 macrophage polarization to activate neuroinflammation, whereas CKAP4 and TLR8 may promote M2 macrophage polarization to alleviate neuroinflammation after ICH, which may provide potential therapeutic targets for ICH.

## Discussion

Given the high morbidity and mortality of stroke, no biomarkers for stroke are available for use in clinical practice, and the identification of potential biomarkers for discriminating between ICH and IS is essential. In this study, we investigated lncRNA and mRNA expression profiles in peripheral blood from ICH patients, IS patients, HTN controls and CTRLs via RNA sequencing. Functional analysis revealed that the most significantly enriched pathway of DEGs after ICH was involved in the immune system response. We further explored the immune cell composition and found that the abundances of 11 types of immune cells, including T-cell subtypes (Th1, Tfh, Th17, Tr1, iTreg, CD4^+^ T, CD8^+^ T and gamma delta T cells), B cells, neutrophils, monocytes and NK cells, were significantly altered after ICH, suggesting that the inflammatory response was involved in neuronal injury after ICH. In addition, by using multiple machine learning algorithms, we established an immune-related biomarker panel (CKAP4, BCL6, and TLR8) whose components are upregulated after ICH. This panel had AUC values of 0.93, 0.95 and 0.82, with sensitivities of 81.3%, 84.4% and 75%, respectively, and specificities of 100%, 96% and 79.7%, respectively, for discriminating ICH patients from HTN controls, CTRLs and IS patients; thus, this panel has potential diagnostic value for ICH.

Functional enrichment revealed that the immune system was the most significantly affected pathway in ICH patients, indicating that the immune system could be a potential therapeutic target related to the pathological effects of ICH. Neutrophils are the first leukocyte population to migrate into the brain after ICH, and their effects appear to be mainly deleterious in the context of brain injury (29). Evidence indicates that anti-polymorphonuclear neutrophil therapy administered via intravenous injection can reduce blood–brain barrier disruption and prevent neurological injury (30, 31). Monocytes also invade within 12 h after ICH, which indicates that they may be involved in secondary injury (29, 32). In our study, neutrophil and monocyte abundances increased during ICH, which is consistent with the findings of previous studies. Suppressed neutrophil and monocyte responses may alleviate neuroinflammation and brain injury, which could be therapeutic targets for ICH.

We also explored the composition of immune cells after ICH and reported that the proportions of T-cell subtypes among blood cells decreased after ICH. Previous studies using ImmuneCellAI analysis also suggested that CD4^+^ T-cell numbers are significantly decreased in ICH patients (33). T-cell subtypes with decreased abundance might be recruited to the brain and involved in inflammatory and anti-inflammatory responses. Reports have suggested that CD4^+^ T-cell numbers increase within 24 h after ICH and that CD8^+^ T-cell numbers increase approximately 3 to 4 days after ICH (34, 35); another study revealed that CD4^+^ T-cell numbers significantly decreased in ICH patients compared with controls, but there was no significant difference in CD8^+^ T-cell numbers between ICH patients and controls (33). There are two subtypes of CD4^+^ T cells, designated Th1 and Th2 cells, on the basis of cytokine secretion patterns (36). Th1 cell differentiation is induced by interleukin-2 and interferon-γ in response to proinflammatory signals (37, 38). Th2 cell differentiation is initiated by IL-4, IL-5 and IL-13, which stimulate B cells to produce abundant antibodies, which are involved in anti-inflammatory functions (39). Studies have demonstrated that Treg cells are beneficial after ICH and IS. Treg cell deficiency increases brain damage and neurological deterioration, and therapeutically increasing Treg cell numbers ameliorates ICH-induced inflammatory injury (40, 41). Transplanting neural stem cells reportedly increases Treg cell numbers and decreases gamma delta T-cell numbers to protect against brain injury in an ICH rat model (42).

Emerging evidence has revealed that machine learning algorithms have been developed to construct diagnostic models, which have become increasingly promising tools for analyzing large amounts of data, such as transcriptome sequences (43, 44). Moreover, specific variation in expression profiles was analyzed by combining GSEA and WGCNA (45) (46). In this study, we identified 18 potential immune-related biomarkers of ICH via GSEA and WGCNA of four groups. After four algorithms were used to select features, 3 overlapping candidate genes (CKAP4, BCL6 and TLR8) were subsequently validated via RT−PCR, and eight machine learning classification algorithms were used to determine their diagnostic value. The AUCs of this panel were 0.93, 0.95 and 0.82 for discriminating ICH patients from HTN controls, CTRLs and IS patients, respectively. CKAP4 is secreted into the serum by tumors and is expected to be a novel serological marker for the diagnosis of various cancers (47). The activation of PI3K–AKT signaling downstream of CKAP4 contributes to immune suppression in macrophages (48). BCL6 modulates the immune response and inflammation by regulating macrophage polarization and plays a critical role in autoimmune encephalomyelitis (49). Toll-like receptor 8 (TLR8) is expressed in different immune cell subtypes and can recognize single-stranded RNA and initiate early inflammatory responses (50). However, the roles of these three genes in ICH patients have not been reported. Our study is the first to show that CKAP4, BCL6 and TLR8 are immune-related biomarkers that differ between ICH patients and IS patients or controls and have promising diagnostic value in patients with ICH. We further found that CKAP4 and TLR8 were downregulated in M1 macrophages and upregulated in M2 macrophages, whereas BCL6 expression was upregulated in M1 macrophages but not in M2 macrophages, indicating that BCL6 may promote M1 macrophage polarization to aggravate neuroinflammation, whereas CKAP4 and TLR8 may promote M2 macrophage polarization to alleviate neuroinflammation after ICH, which may be potential therapeutic targets for ICH.

There are some limitations of our study. First, we focused on one timepoint after ICH, and multiple time points for multilayer distribution analysis should be used to control for confounding factors. A larger multicenter study with more individuals should be conducted for external validation. Second, to ensure the accuracy of the identified RNA panel in guiding clinical treatment, single cells need to be isolated and analyzed via single-cell RNA sequencing, and the functions and mechanism of the anti-inflammatory effects of CKAP4 and TLR8, as well as the proinflammatory effects of BCL6 in ICH patients, need to be further explored in future studies. Third, the eight machine learning classifiers were applied to small training datasets because of the limited sample size; however, the best performing classifier was comprehensively validated and then confirmed with a series of indices. Therefore, the selected features are considered significant.

## Conclusion

Our study comprehensively compares the transcriptome profiles of ICH patients, IS patients, HTN controls and CTRLs to aid in the early differentiation of patients with ICH from those with IS. Eleven immune cell types with significantly altered abundance after ICH were identified; these findings might provide useful insight into the pathogenesis and therapeutic approaches for patients with ICH. Furthermore, an RNA panel (CKAP4, BCL6, and TLR8) was developed as a potential biomarker for ICH detection. This study provides a new perspective on the pathogenesis of ICH and a more effective diagnostic tool.

## Data Availability

The datasets presented in this study can be found in online repositories. The names of the repository/repositories and accession number(s) can be found below: HRA001807 (GSA; https://ngdc.cncb.ac.cn/gsa-human/browse/HRA001807).
