# Supplementary material for: Identification of immune-related biomarkers for intracerebral hemorrhage diagnosis based on RNA sequencing and machine learning
Source: Front Immunol. 2024 Aug 30;15:1421942. doi: 10.3389/fimmu.2024.1421942 (PMC11392791; doi:10.3389/fimmu.2024.1421942)
Supplement: Supplementary file 1 [file DataSheet1.docx]

**Supplementary Materials**

1. **Supplementary Figures**

**
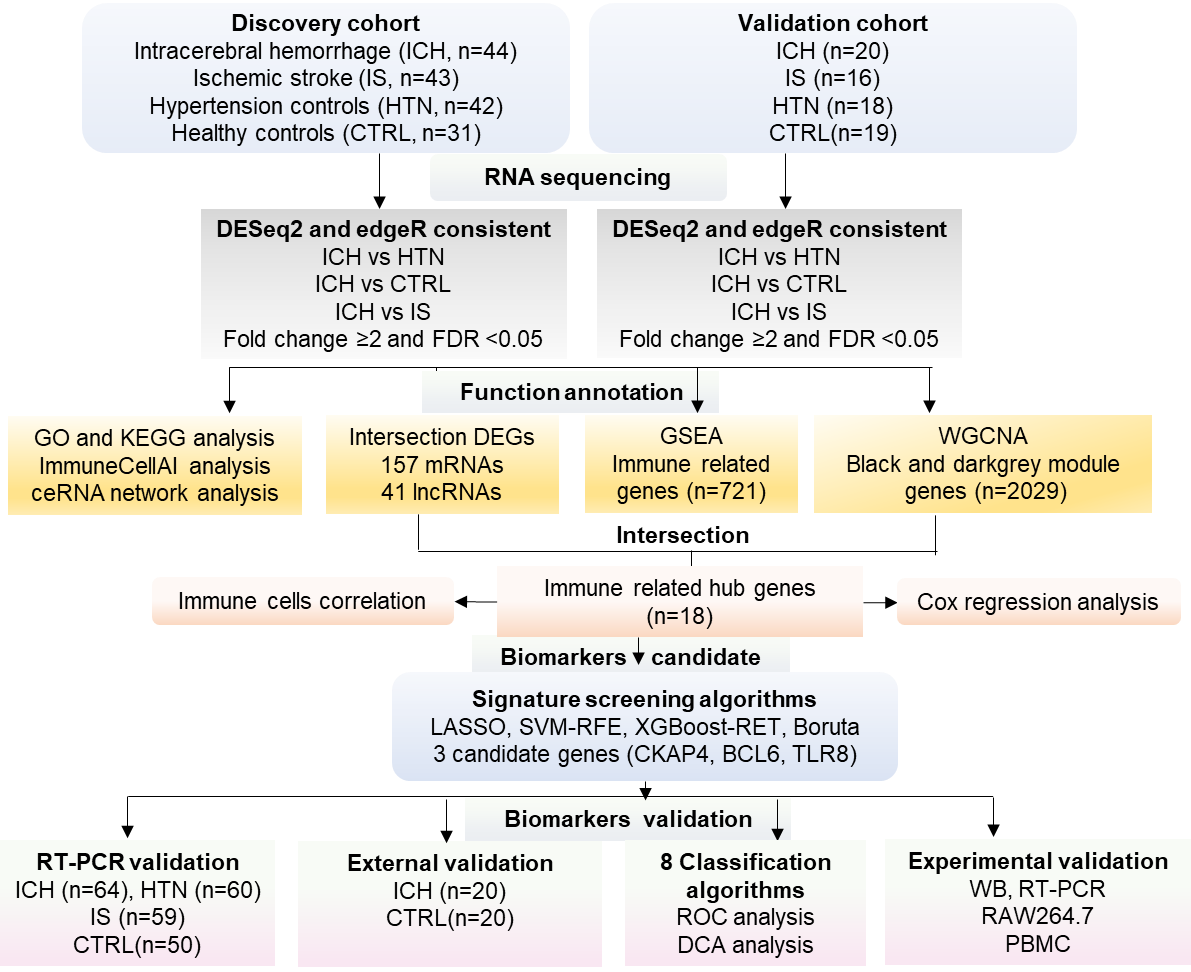
**

**Supplemental Figure 1. Work flow.** The diagram of the data analysis process in this study. ICH: intracerebral hemorrhage; HTN: hypertension; IS: ischemic stroke; CTRL: healthy control. FC: Fold change; FDR: False discovery rate.


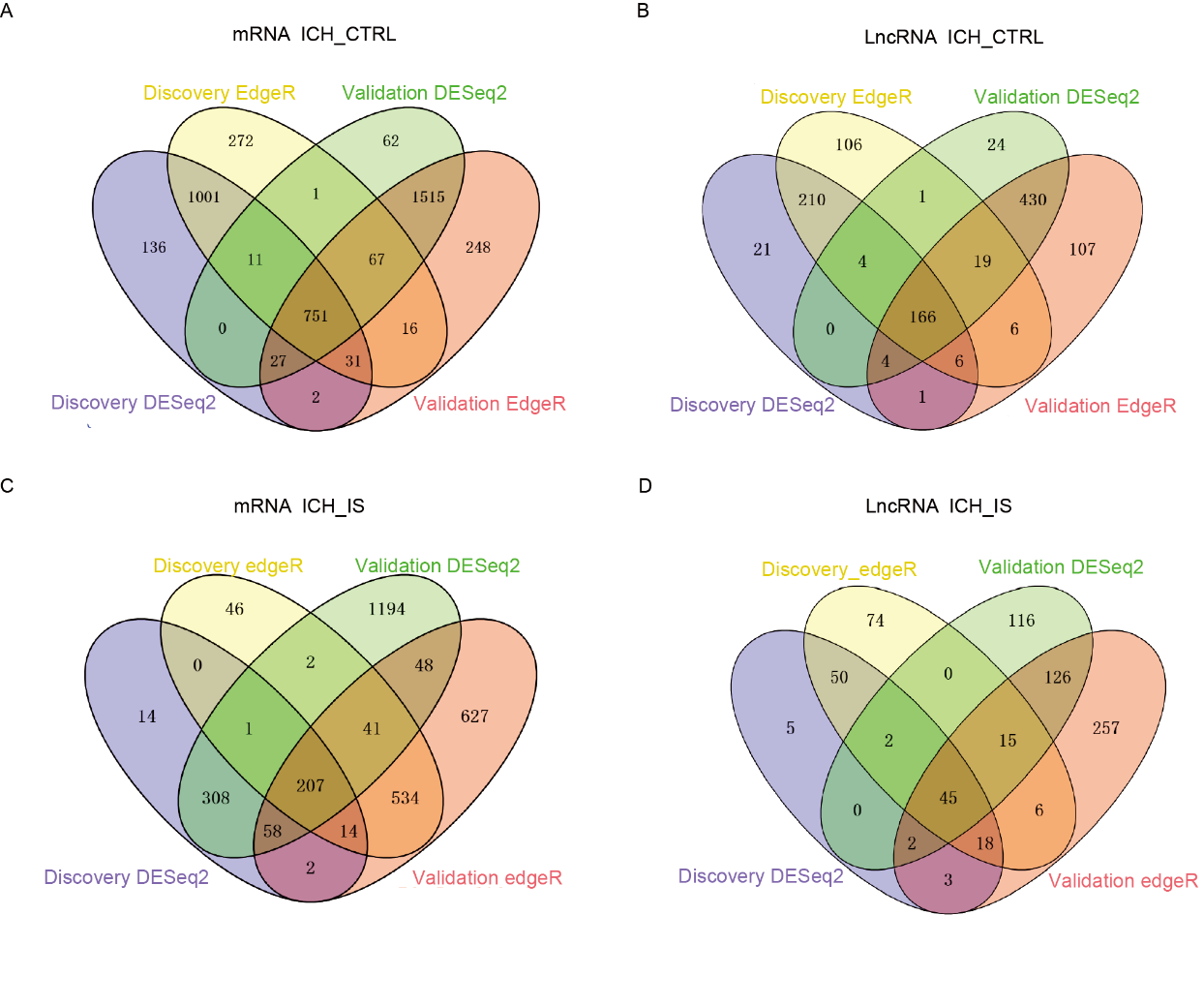


**Supplemental Figure 2. Venn diagram showing the consistently altered mRNAs (A, C) and lncRNAs (B, D) in ICH patients compared with CTRLs (A, B) and ICH patients compared with IS patients (C, D) in the discovery and validation cohorts with both the DESeq2 and edgeR methods.** ICH: intracerebral hemorrhage; HTN: hypertension; IS: ischemic stroke; CTRL: healthy control. Statistical significance levels were corrected for multiple testing using the Benjamini–Hochberg procedure.


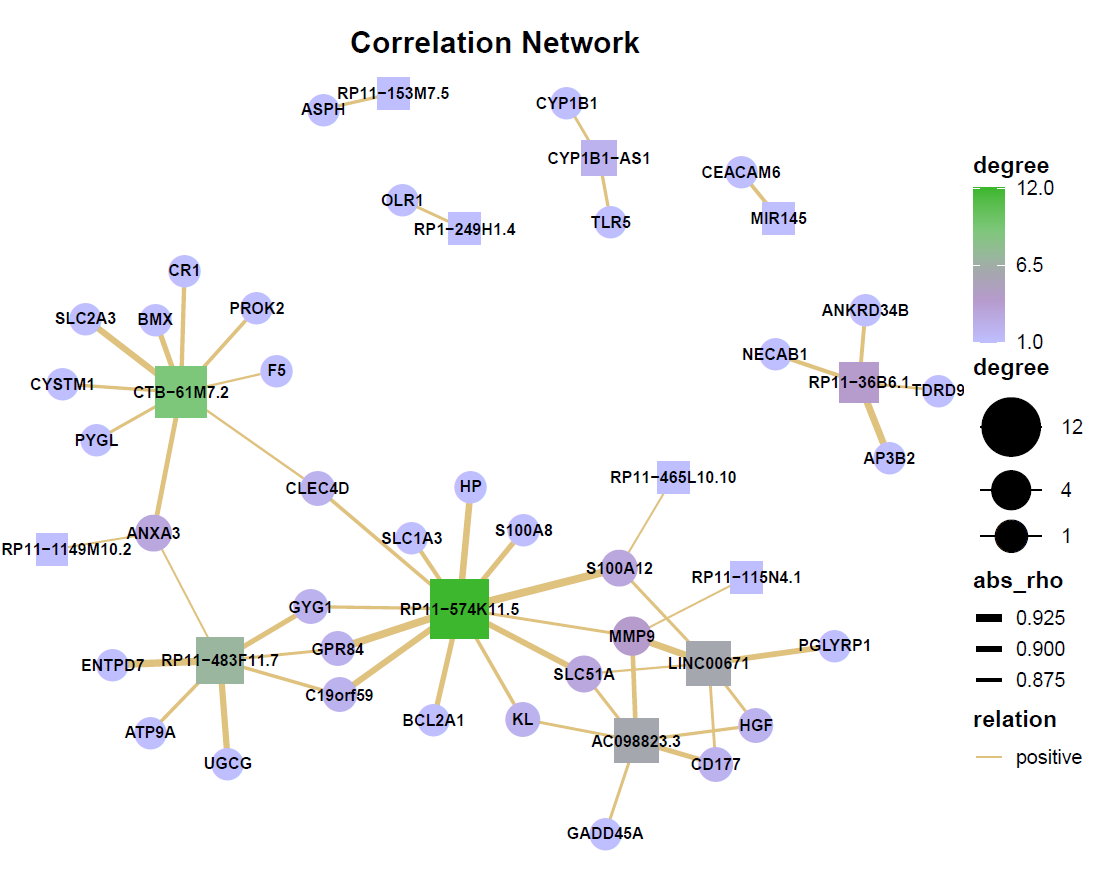


**Supplemental Figure 3. The lncRNA-mRNA coexpression network.** The circles represent mRNAs, and the squares represent lncRNAs; Shape size represents degree; the thickness of the lines represent the size of the absolute value of rho.


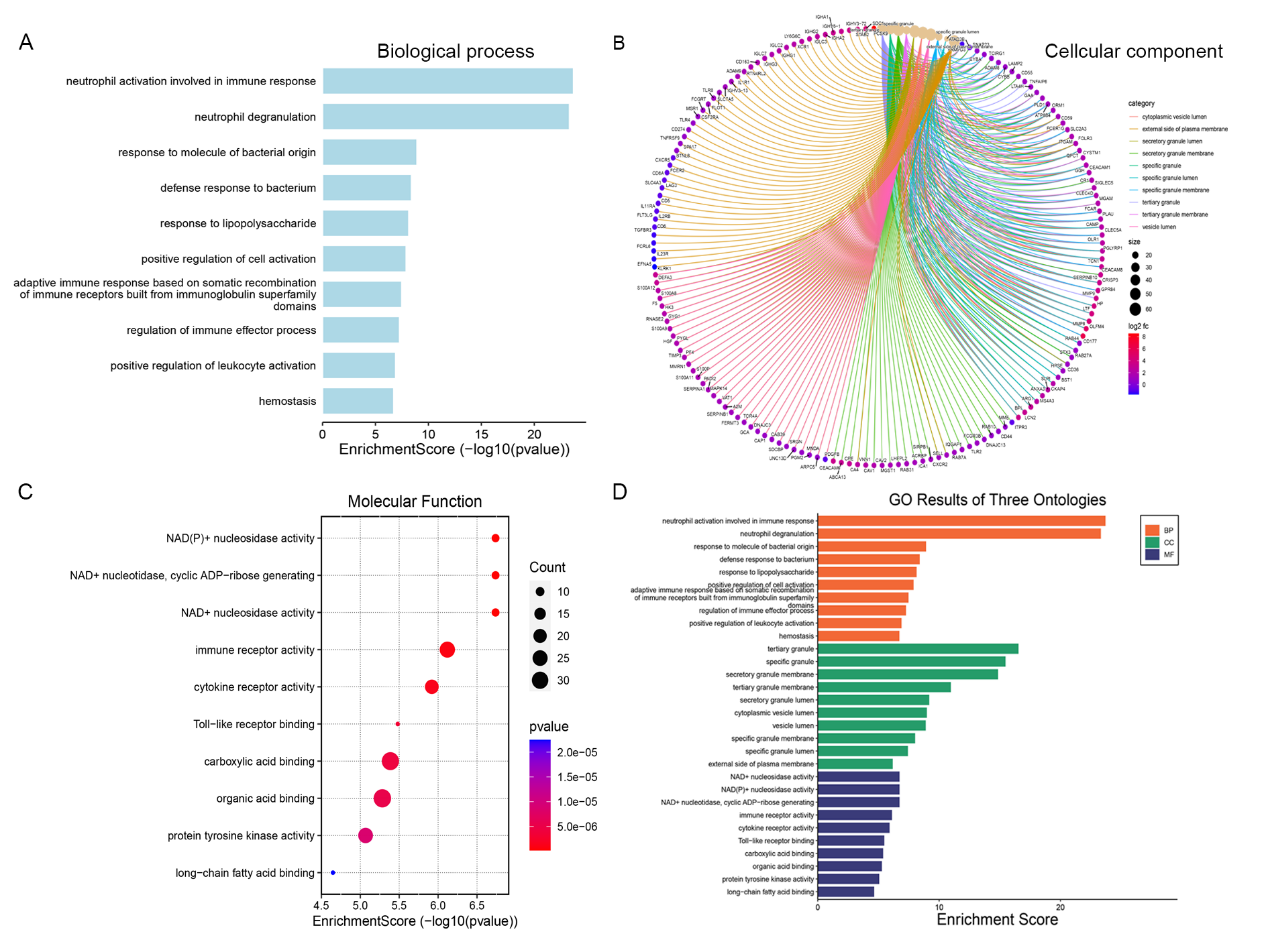


**Supplemental Figure 4. Gene Ontology (GO) pathway analyses of significantly altered mRNAs.** The top 10 terms of biological process (**A, D**), cell component **(B, D)** and molecular function **(C, D)** from GO enrichment analysis of differentially expressed genes. GO: Gene ontology. Statistical significance levels were corrected for multiple testing using the Benjamini–Hochberg procedure.

**
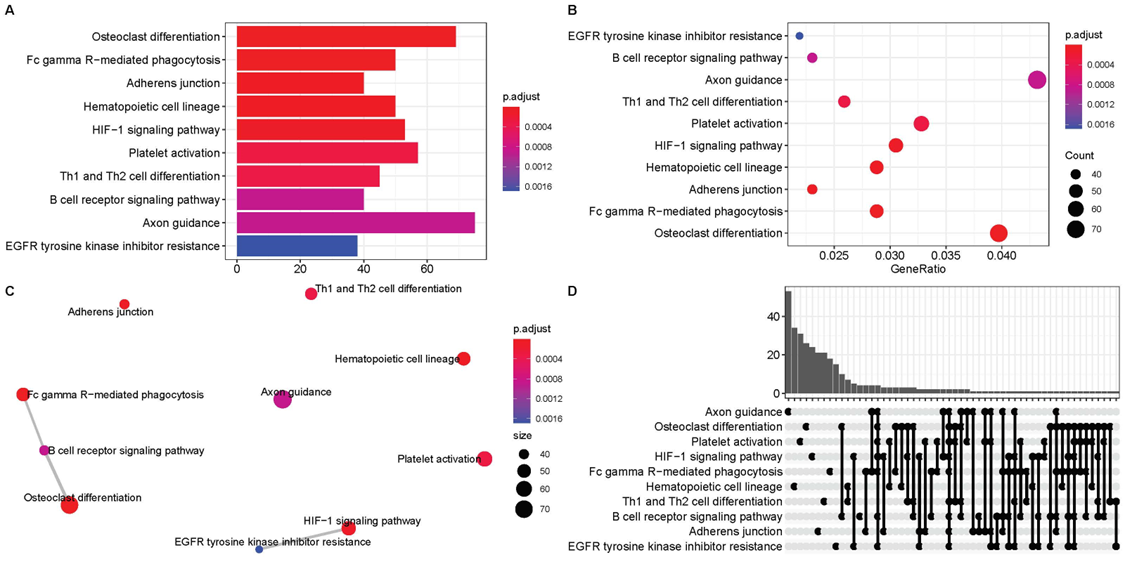
**

**Supplemental Figure 5. The representative pathways with significant differences of genes in ICH based on GSEA.** (**A**) Enrichment plot, bubble diagram **(B)**, network diagram **(C)** and upset plot **(D)** show the top 10 pathways in annotated gene sets. Statistical significance levels were corrected for multiple testing using the Benjamini–Hochberg procedure.

**
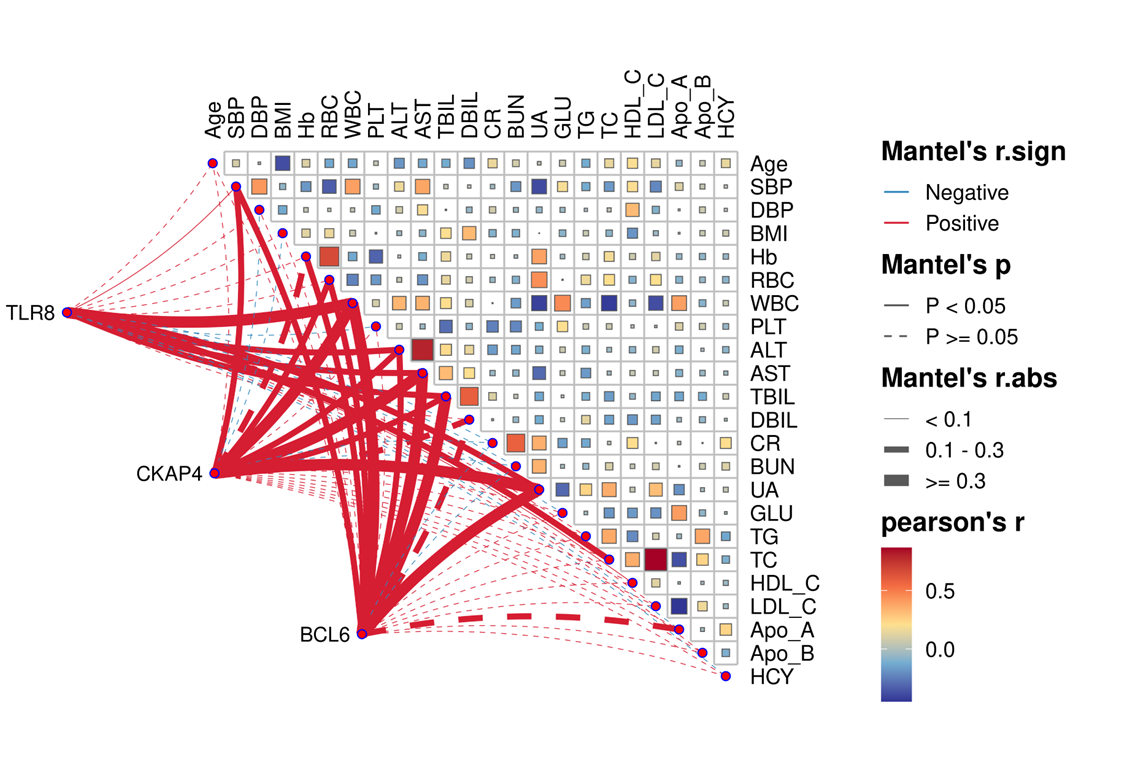
Supplemental Figure 6. Correlation between three candidate genes and clinical characteristic levels in ICH patients.**

1. **Supplementary Tables**

**Supplementary Tables 1-6 refer to Supplemental Excel file.**

**Supplementary Table 1. Consistently differentially Expressed mRNAs in ICH compared to HTN** **by the DESeq2 and edgeR method in both 2 cohorts.**

**Supplementary Table 2. Consistently differentially Expressed lncRNAs in ICH compared to** **HTN** **by the DESeq2 and edgeR method in both 2 cohorts.**

**Supplementary Table 3. Consistently differentially Expressed mRNAs in ICH compared to CTRL by the DESeq2 and edgeR method in both 2 cohorts.**

**Supplementary Table 4. Consistently differentially Expressed lncRNAs in ICH compared to CTRL by the DESeq2 and edgeR method in both 2 cohorts.**

**Supplementary Table 5. Consistently differentially Expressed mRNAs in ICH compared to IS patients by the DESeq2 and edgeR method in both 2 cohorts.**

**Supplementary Table 6. Consistently differentially Expressed lncRNAs in ICH compared to IS patients by the DESeq2 and edgeR method in both 2 cohorts.**

**Supplementary Table 7. LncRNA-mRNA coexpression pairs.**

**Supplemental Table 8. Demographics and characteristics of the external validation cohorts**

|  | Control  （n=20） | ICH  (n = 20) | *P*-value |
| --- | --- | --- | --- |
| Age, y | 58.7±13.6 | 61.3±9.1 | 0.972 |
| Men, % | 10 (50.0) | 10 (50.0) | 0.999 |
| BMI, kg/m^2^ | 24.6±3.3 | 24.5±3.8 | 0.582 |
| SBP, mmHg | 128.4±10.7 | 139.3±5.1 | ＜0.01 |
| DBP, mmHg | 78.4±5.3 | 81.1±9.3 | 0.029 |
| HDL-C, mmol/L | 1.4±0.3 | 1.3±0.3 | 0.221 |
| LDL-C, mmol/L | 3.1±1.1 | 2.8±0.9 | 0.410 |
| TC | 4.8±1.2 | 4.5±1.0 | 0.320 |
| TG, mmol/L | 1.4±0.8 | 1.5±0.7 | 0.660 |
| Glucose, mmol/L | 5.8±1.3 | 6.8±3.5 | 0.249 |
| Smoking, % |  |  | 0.659 |
| Never | 13 (65.0） | 11 (55.0) |  |
| Former | 2 (10.0) | 4 (20.0) |  |
| Current | 5 (25.0) | 5 (25.0) |  |
| Drinking, % |  |  | 0.288 |
| Nondrinker | 16 (80.0) | 13 (65.0) |  |
| Drinker | 4 (20.0) | 7 (35.0) |  |

Data is expressed as mean ± standard deviation or n (%). BMI: Body mass index; SBP: Systolic blood pressure; DBP: Diastolic blood pressure; TC: Total cholesterol; TG: Triacylglycerol; HDL-C: High-density lipoprotein cholesterol; LDL-C: Low-density lipoprotein cholesterol; GLU, Glucose; ICH, Intracerebral hemorrhage. Statistical comparisons for percentages were performed using chi-square test. Comparisons between means or medians were performed using 2-tailed Student’s *t* test.

**Supplementary Table 9. Sequences of real-time PCR primers**

| Primer Name | Sequence (5’-3’) |
| --- | --- |
| Mus-TNF-α-F | CCTGTAGCCCACGTCGTAG |
| Mus-TNF-α-R | GGGAGTAGACAAGGTACAACCC |
| Mus-GAPDH-F | TGACCTCAACTACATGGTCTACA |
| Mus-GAPDH-R | CTTCCCATTCTCGGCCTTG |
| Mus-CD206-F | CTCTGTTCAGCTATTGGACGC |
| Mus-CD206-R | TGGCACTCCCAAACATAATTTGA |
| Mus-Arginase-1-F | GGTGGCAGAGGTCCAGAAGAA |
| Mus-Arginase-1-R | GAGTGTTGATGTCAGTGTGAGCA |
| Mus-iNOS-F | CAGCTGGGCTGTACAAACCTT |
| Mus-iNOS-R | CATTGGAAGTGAAGCGTTTCG |
| Mus-Cxcl10-F | GGATGGCTGTCCTAGCTCTG |
| Mus-Cxcl10-R | TGAGCTAGGGAGGACAAGGA |
| Mus-BCL6-F | CCGGCACGCTAGTGATGTT |
| Mus-BCL6-R | GCACTGTCTTATGGGCTCTAAAC |
| Mus-CKAP4-F | GGGCTGGTATGTCCATCACG |
| Mus-CKAP4-R | ATCCCTCTGACGGGAGAAGTC |
| Mus-TLR8-F | TTCCTCACATTCCTTACCACCTC |
| Mus-TLR8-R | GTGATAGATAAACCAAACATCCCAG |
| Human-GAPDH-F | TGAACGGGAAGCTCACTGG |
| Human- GAPDH-R | TCCACCACCCTGTTGCTGTA |
| Human-BCL6-F | GGAGTCGAGACATCTTGACTGA |
| Human-BCL6-R | ATGAGGACCGTTTTATGGGCT |
| Human-CKAP4-F | TGGACAGTTTGGTTGCATACTC |
| Human-CKAP4-R | CCTCAGGTCATCTAGTAAACCCT |
| Human-TLR8-F | ATGTTCCTTCAGTCGTCAATGC |
| Human-TLR8-R | TTGCTGCACTCTGCAATAACT |
